# The diversity and evolutionary relationships of ticks and tick-borne bacteria collected in China

**DOI:** 10.1186/s13071-022-05485-3

**Published:** 2022-10-01

**Authors:** JunHua Tian, Xin Hou, MiHong Ge, HongBin Xu, Bin Yu, Jing Liu, RenFu Shao, Edward C. Holmes, ChaoLiang Lei, Mang Shi

**Affiliations:** 1 Hubei Key Laboratory of Resources Utilization and Sustainable Pest Management, College of Plant Science and Technology, Huazhong Agricultural University, Wuhan, Hubei Province 430070 China; 2 School of Medicine, Shenzhen Campus of Sun Yat-Sen University, Sun Yat-Sen University, Shenzhen, Guangdong Province 518107 China; 3 Wuhan Centers for Disease Control and Prevention, Wuhan, Hubei Province 430015 China; 4 Wuhan Academy of Agricultural Sciences, Wuhan, Hubei Province 430345 China; 5 Jiangxi Province Center for Disease Control and Prevention, Nanchang, Jiangxi Province 330029 China; 6 School of Science, Technology and Engineering, University of the Sunshine Coast, Sippy Downs, QLD 4558 Australia; 7 GeneCology Research Centre, University of the Sunshine Coast, Sippy Downs, QLD 4558 Australia; 8 Sydney Institute for Infectious Diseases, School of Life & Environmental Sciences and School of Medical Sciences, The University of Sydney, Camperdown, NSW 2006 Australia

**Keywords:** Ticks, Metagenomics, Bacteria, Evolution, Phylogeny, Co-divergence

## Abstract

**Background:**

Ticks (order Ixodida) are ectoparasites, vectors and reservoirs of many infectious agents affecting humans and domestic animals. However, the lack of information on tick genomic diversity leaves significant gaps in the understanding of the evolution of ticks and associated bacteria.

**Results:**

We collected > 20,000 contemporary and historical (up to 60 years of preservation) tick samples representing a wide range of tick biodiversity across diverse geographic regions in China. Metagenomic sequencing was performed on individual ticks to obtain the complete or near-complete mitochondrial (mt) genome sequences from 46 tick species, among which mitochondrial genomes of 23 species were recovered for the first time. These new mt genomes data greatly expanded the diversity of many tick groups and revealed five cryptic species. Utilizing the same metagenomic sequence data we identified divergent and abundant bacteria in *Haemaphysalis*, *Ixodes*, *Dermacentor* and *Carios* ticks, including nine species of pathogenetic bacteria and potentially new species within the genus *Borrelia*. We also used these data to explore the evolutionary relationship between ticks and their associated bacteria, revealing a pattern of long-term co-divergence relationship between ticks and *Rickettsia* and *Coxiella* bacteria.

**Conclusions:**

In sum, our study provides important new information on the genetic diversity of ticks based on an analysis of mitochondrial DNA as well as on the prevalence of tick-borne pathogens in China. It also sheds new light on the long-term evolutionary and ecological relationships between ticks and their associated bacteria.

**Graphical Abstract:**

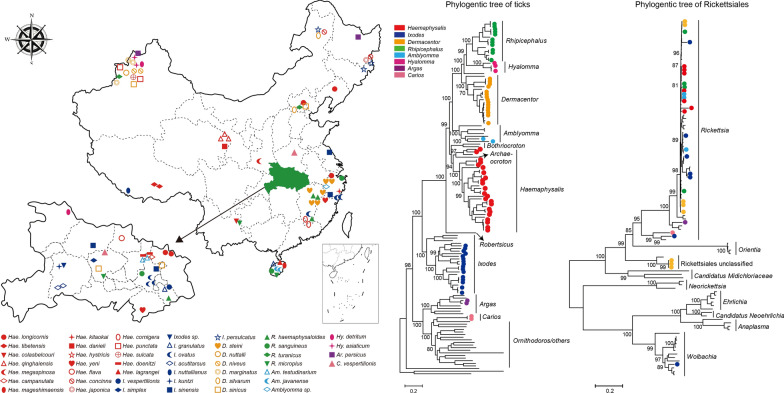

**Supplementary Information:**

The online version contains supplementary material available at 10.1186/s13071-022-05485-3.

## Background

Ticks (Acari: Ixodidae) are hematophagous arthropods and act as vectors for various infectious pathogens. Ticks are classified into three families: Argasidae (soft ticks), Ixodidae (hard ticks) and Nuttalliellidae. While there is only one extant species in the Nuttalliellidae, considered the closest extant relative to the ancestral tick lineage, > 700 and 200 recognized species have been identified within the Ixodidae and Argasidae, respectively [1–3].

China covers a large geographic area and possesses a variety of ecosystems. To date, at least 125 tick species from nine genera have been reported across 34 provinces in China, representing 13.9% of tick species identified globally [4]. The most frequently reported species in China are *Haemaphysalis longicornis*, *Dermacentor silvarum*, *Ixodes persulcatus*, *Haemaphysalis conicinna*, *Rhipicephalus microplus* and *Rhipicephalus sanguineus *sensu lato [5, 6]. *Dermacentor sinicus*, *Ixodes sinensis*, *Haemaphysalis tibetensis* and *Haemaphysalis qinghaiensis* have been only reported in China, although there is a lack of genomic data for these species [7].

Mitochondrial (mt) genomes are widely used in molecular systematics because their relatively high rate of evolutionary change provides greater phylogenetic resolution at the genus or family levels [8]. For ticks, complete mt genomes representing 66 species from 18 genera have been sequenced to date [9, 10]. In most arthropods, tick mt genomes are circular, 14–16 kb in length, and contain 37 genes, including 13 protein coding genes, 22 tRNA genes and two rRNA genes. Phylogenetic analyses based on protein coding and rRNA gene sequences show great consistency in genus level classification [9]. A single genome re-arrangement event has been identified in the Ixodidae, including the genera *Rhipicephalus*, *Dermacentor*, *Amblyomma* and *Haemaphysalis*, which possess a translocation of tRNA genes (*trnL*_*1*_, *trnL*_*2*_, *trnC*) and an inversion of the *trnC* gene [11, 12].

Ticks have long been regarded as disease vectors, with an increasing number of human disease associations described in recent years [13–16]. Since 1982, more than 30 emerging tick-borne disease agents have been identified from at least 28 tick species, causing a variety of human infections including rickettsioses, Q fever and borrelioses. Together, these have accounted for nearly 50,000 cases per year since 2013 as reported by National Notifiable Disease Surveillance System in the USA [17, 18]. Among these, rickettsial disease and Q fever are caused by two groups of obligate intracellular endosymbiont bacteria in ticks: the spotted fever group (SFG) rickettsiae [19, 20] and *Coxiella burnetii* [21], harbored by hard and soft ticks, respectively. In contrast, borrelioses, represented by Lyme disease and relapsing fever, are mainly caused by bacteria in the genus *Borrelia* (phylum Spirochaetes). Lyme borreliosis (LB) species (*B. burgdorferi*) are generally carried by *Ixodes* ticks, whereas the relapsing fever *Borrelia* group is generally carried by *Ornithodoros sonrai*, *Ornithodoros erraticus* and *Ornithodoros moubata* [22, 23]. In addition to known pathogens, ticks harbor a number of other bacterial symbionts in the genera *Rickettsia* and *Coxiella*. While their public health significance remains unclear, these microbes are highly prevalent in tick species and can be transmitted transovarially [24–27].

Despite growing interest in tick-borne pathogens, our knowledge of the genetic diversity of ticks in China and the bacteria they carry is limited to a small number of common species. Herein, we collected and sequenced 46 tick species representing the biodiversity of ticks across China. For the first time to our knowledge, we revealed the genetic diversity of both ticks and their bacterial symbionts, enabling a more systematic study of their co-evolutionary history.

## Methods

### Sample collection

**Fig. 1:**
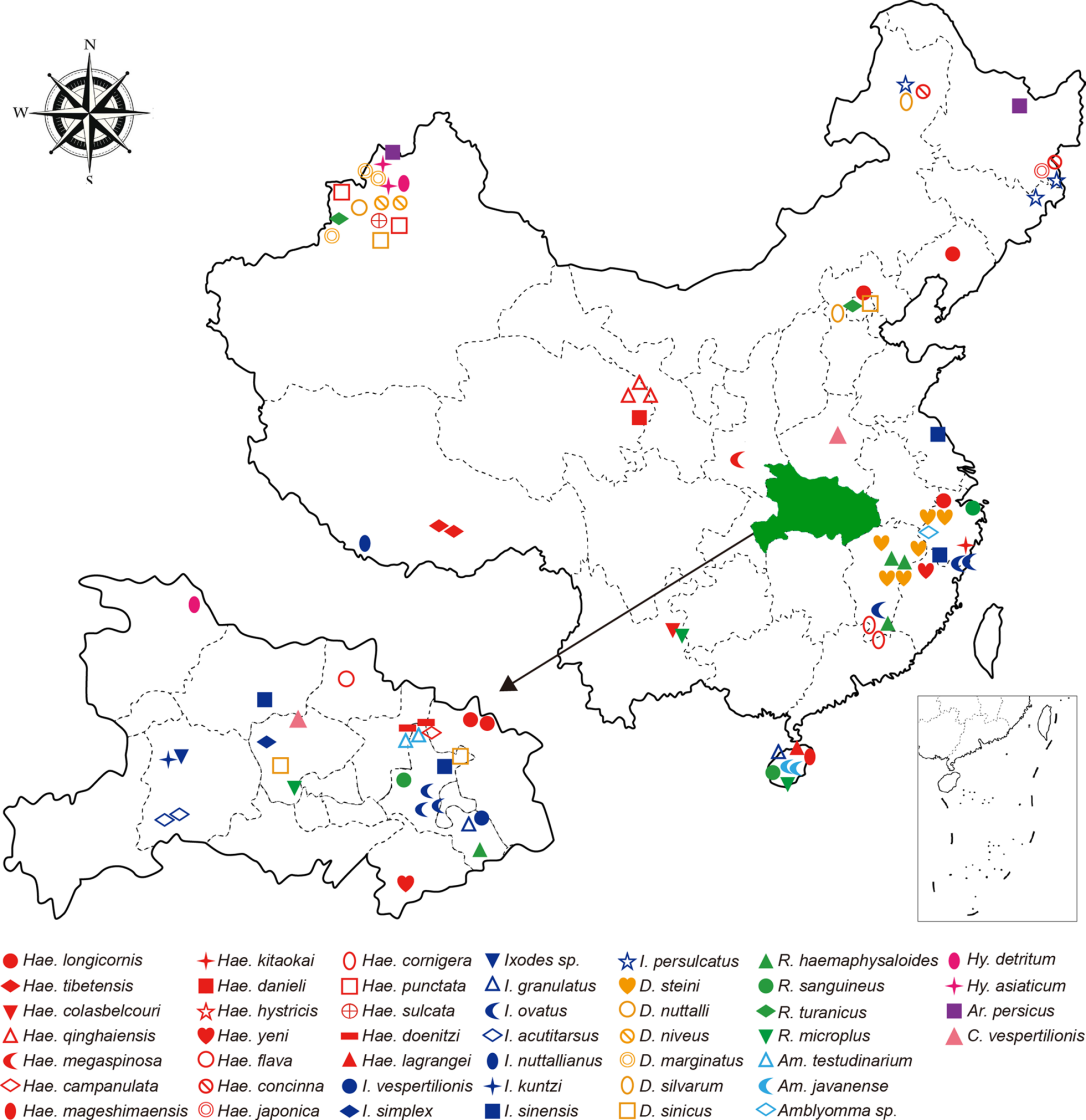
Sampling locations of 46 tick species collected in China. The map of Hubei province (shaded green) was magnified for clarity. We use different colors and shapes to represent 46 tick species from eight genera: *Haemaphysalis* (red), *Ixodes* (dark blue), *Dermacentor* (yellow), *Rhipicephalus* (green), *Amblyomma* (sky blue), *Hyalomma* (magenta), *Argas* (purple) and *Carios* (pink)

Between 1959 and 2019 > 20,000 ticks belonging to eight genera (*Rhipicephalus, Hyalomma, Dermacentor, Amblyomma, Haemaphysalis, Ixodes, Argas* and *Carios*) were collected from different geographic regions in China (Fig. 1, Additional file 1: Table S1). Most ticks were directly picked from infested wild and domestic animals, although some were captured using a drag-flagging method. Upon capture, each tick was sexed and morphologically identified to species by trained field biologists and further confirmed by sequencing mitochondrial genome sequences. Among these samples, 42% (*I. simplex*, *A. testudinarium* and *Hae. qinghaiensis*) were preserved in alcohol at room temperature for 1–60 years, while others were captured alive and stored at the temperature of − 80 ℃ until DNA extraction.

### DNA extraction and sequencing

For most of the samples, we used a direct total DNA extraction and sequencing approach. For each sample, the ticks, including questing and partially engorged ticks, were first washed three times in PBS solutions before they were homogenized using the Mixer mill MM400 (Restsch). The homogenates were then subject to DNA extraction using the QIAamp DNA Mini Kit (Qiagen) and Mollusc DNA Kit D3373 (OMEGA) following the manufacturer’s recommendations. Library preparations were performed by BGI · Tech and/or Novogene and then 150 bp pair-end sequencing on an Illumina HiSeq 4000 platform.

For two of the libraries (A3 and A4), the mitochondria were first purified from cell homogenates before DNA extraction. Library preparation and sequencing were performed as described above. To purify the mitochondria, cell homogenates were first subjected to low-speed centrifugation (10 min at 100 *g*) for insoluble debris removal. The supernatant was further centrifuged at 10,000 *g* for 5 min to obtain the mitochondria. The total sequenced data generated varied from 0.2 to 30.1 Gbp. For some of the libraries (A41, C7, D23 and C23) where mitochondrial genome received partial coverage, we increased the sequencing depth to 4.5 to 12.61 Gbp to obtain better coverage.

### Mitochondrial genome assembly and phylogenetic analyses

Clean sequence reads were assembled de novo using MEGAHIT version 1.2.6 [28] with default parameters. To obtain mt genome sequences, the assembled contigs were compared against reference mt genomes in GenBank using blastn [29]. We set the *E*-value to 1E-10 to maintain high sensitivity and a low false-positive rate. Contigs with unassembled overlaps were merged to form longer mt contigs using the SeqMan program implemented in the Lasergene software package version 7.1 [30]. We used Bowtie2 [31] for confirmation and/or extension of the mt genomes. The protein-coding genes and rRNA genes of the assembled mt genomes were annotated using the Spin program implemented in the Staden package [32]. The annotated mt genomes of these ticks were deposited in GenBank under accession numbers OM368258—OM368330 and MK344649.

To infer phylogenetic trees, we used 74 mt genomes generated in this study plus an additional 62 reference tick mt genomes obtained from GenBank. Individual alignments were performed on each of the 13 mt protein coding genes (*ATP6, ATP8, COX1, COX2, COX3, CYTB, NAD1, NAD2, NAD3, NAD4, NAD4L, NAD5, NAD6*) and two rRNA genes (*12S rRNA* and *16S rRNA*). Protein coding genes were aligned based on codons using the ClustalW implemented in MEGA version 5.2 [33]. Ambiguously aligned regions were removed. Two rRNA genes were aligned using the MAFFT version 7.4 [34] employing the L-INS-i algorithm with all ambiguous aligned regions removed using TrimAL version 1.2rev59 [35]. Individual gene alignments were then concatenated to form super-alignments for subsequent phylogenetic analysis. These comprised: an (i) “all gene” data set containing 13 protein coding genes and two rRNA genes and (ii) “a protein coding gene” data set that only contained the 13 protein coding genes. All data sets were analyzed using both maximum likelihood (ML) and Bayesian algorithms implemented in IQ-TREE version 1.6.12 [36] and MrBayes version 3.2 [37], respectively. For ML analyses, the best-fit nucleotide substitution model was selected using ModelFinder [38], and tree topologies were evaluated with an ultrafast bootstrap approximation approach [39] with 1000 replicates. For Bayesian tree inference, we used the substitution model as described above with 1,000,000 generations in two runs, and each was sampled every 500 generations with a burn-in of 25%.

### Identification and characterization of bacterial pathogens in ticks

The initial taxonomy profiling was performed by metaphlan2 [40]. Furthermore, for each library, we searched for the existence of marker genes of Rickettsiales (*groEL*, *gltA*), *Borrelia* (*groEL, bamA, flaB*) and *Coxiella* (*groEL, rpoB*). The assembled contigs were compared against the database of reference marker genes downloaded from GenBank using blastx, with an *E*-value cut-off set at 1E-10. For libraries with incomplete marker gene coverage, partial gene sequences were obtained by first mapping reads to a reference marker gene sequence of bacterial pathogens using Bowtie2 [31] and generating consensus sequences from the mapped reads. The clean reads were subsequently mapped to *groEL* gene sequences to estimated gene abundance as the number of mapped reads per million total reads (RPM, RPM of a gene = Number of reads mapped to a gene × 10^6^/Total number of mapped reads from given library). Based on these genes, we then compared the bacterial pathogens identified in this study with those previously described by estimating phylogenetic relationships using the ML and Bayesian methods described above.

### The co-phylogenetic relationship between ticks and their bacterial pathogens

We used the BaTS (Bayesian tip-association significance testing) program [41] to test whether bacteria pathogens (*Rickettsia* and *Coxiella*) form close co-phylogenetic relationship with their tick hosts. This analysis considered tick host and pathogen phylogenies at the genus level: that is, *Rhipicephalus*, *Hyalomma, Dermacentor, Amblyomma, Haemaphysalis, Ixodes, Argas* and *Carios*. Specifically, we estimated the Association Index [42] and compared it with a null distribution generated using 1000 replicates of state randomization (i.e. tick genera) across a credible set of pathogen trees generated by MrBayes version 3.2 [37] as described above.

To examine the extent of bacteria-tick co-divergence, we performed event-based co-phylogenetic reconstructions using the Jane program, version 4.0 [43]. The ‘cost’ scheme for analyses in Jane was set as follows: co-divergence = 0, duplication = 1, host switch = 1, loss = 1, failure to diverge = 1. The number of generations and the population size were both set to 100. The significance of co-divergence was derived by comparing the estimated costs to null distributions calculated from 100 randomizations of host tip mapping. In addition, we performed a distance-based analysis to test the hypothesis of bacterial-tick co-divergence using ParaFit as implemented in the COPYCAT software package version 2.0 [44], comparing distance matrices derived from the bacteria and tick host phylogenies. Significance testing was based on 9999 randomizations of the association matrices. Additionally, to visualize the association between bacteria and their tick hosts, a tanglegram was generated by matching each bacterial species to their associated ticks using TreeMap version 3.0beta [45].

### The influence of geographic and tick genetic distance on pathogen genetic diversity

Bacterial and tick genetic distance matrices were derived from pairwise genetic comparisons using MEGA version 5.2 [33]. Geographic distances (Euclidean distance) were calculated using spatial coordinates of the samples derived from information on their geographic location. We used Mantel correlation analysis [46] to test the extent of the correlation between these matrices. Both a simple Mantel’s test and partial Mantel’s test were performed, and the correlation was evaluated using 10,000 permutations. To access which of the two factors—geographic or tick genetic distances—best explained total variation in the bacteria genetic distance matrices, we performed a multiple linear regression analysis [47] on these distance matrices. The statistical significance of each regression was evaluated by performing 1000 permutations. All statistical analyses were performed using the Ecodist package implemented in R 3.4.4 [48], and all statistical results were considered significant at a *P*-value of 0.05.

## Results

### Morphological identification and characterization of ticks

Between 1959 and 2019, more than 20,000 ticks were collected from a wide range of hosts (e.g. cattle, goats, camels, hedgehogs) across China (Fig. 1, Table 1, Additional file 1: Table S1). Species identification of hard ticks was carried out based on morphological characters, such as palps, basis capituli, cornuae, auriculae, dentition formula, punctuations, coxal spurs, amongst others [49–51]. Identification of soft ticks was conducted using taxonomic keys proposed by Hoogstraal [54], Teng [50] and Sun et al. [52–54]. This revealed a total of at least 46 species comprising two families (Ixodidae and Argasidae) and eight genera: *Haemaphysalis* (19), *Ixodes* (10), *Dermacentor* (six), *Rhipicephalus* (four), *Hyalomma* (two), *Amblyomma* (three), *Argas* (one) and *Carios* (one). Among these genera are the six most common tick species in China [6]: *R. microplus*, *R. sanguineus* s.l., *I. persulcatus*, *Hae. longicornis*, *D. silvarum* and *Hyalomma asiaticum*. In addition, other common species were collected and analyzed, including *I. sinensis* (vector of *B. burgdorferi* [55]), *Ixodes ovatus*, *D. steini*, *Haemaphysalis yeni*, *Hae. concinna*, *Hyalomma scupense*, *Rhipicephalus turanicus, Rhipicephalus haemaphysaloides* and *Argas persicus* from at least two provinces. In comparison, other species were more locally defined, such as *Hae. tibetensis* from Tibet, *Hae. qinghaiensis* and *Haemaphysalis danieli* from Qinghai, *Hy. asiaticum*, *Haemaphysalis punctata*, *Dermacentor marginatus* and *Dermacentor niveus* from Xinjiang, *Ixodes acutitarsus* from Hubei and *Haemaphysalis lagrangei* and *Haemaphysalis mageshimaensis* from Hainan. In addition, we obtained a number of very rare species, particularly *Amblyomma javanense*, *Ixodes simplex*, *Ixodes nuttallianus*, *Ixodes crenulatus*, *Ixodes kuntzi*, *Haemaphysali kitaokai* and *Carios vespertilionis*, some of which were collected from wildlife animals including bats, pangolins and flying squirrels (Pteromyini). Other samples were collected by drag-flagging methods or were historical samples preserved in ethanol for > 60 years. For example, the oldest sample from our data set (*A. javanense*) was collected in the 1960s from a wild Chinese pangolin (*Manis pentadactyla*). In the case of two samples (i.e. C20, A29), identification could only take place to the genus level, in *Amblyomma* and *Ixodes*, respectively. Since we were unable to identify them at the species level using morphological characteristics, they were tentatively assigned as potential new species.

**Table 1:** Sampling locations, animal hosts and bacterial pathogens of tick species in China at the genus level

Species(genus)	Location	Host	No. of libraries	Pathogen
*Rhipicephalus*	Hubei, Jiangxi, Yunnan, Beijing, Zhejiang, Xinjiang, Hainan	Cattle, dog, hedgehog	12 (11)	*Rickettsia, Coxiella,*
*Hyalomma*	Xinjiang, Hubei	Cattle, goat, camel	4 (3)	–
*Dermacentor*	Hubei, Jiangxi, Inner Mongolia, Beijing, Hebei, Zhejiang, Xinjiang	Cattle, goat, rabbit, hedgehog, wild boar	18 (15)	*Rickettsia, Coxiella,* Rickesiales unclassified
*Amblyomma*	Hubei, Zhejiang, Hainan	Wild boar, Malaysia pangolin, Chinese pangolin	6 (2)	*Rickettsia*
*Haemaphysalis*	Hubei, Jiangxi, Fujian, Zhejiang, Hainan, Yunnan, Beijing, Shaanxi, Heilongjiang, Liaoning, Qinghai, Tibet, Xinjiang, Inner Mongolia	Cattle, yak, pheasant, dog, hedgehog, muntjac, goat, wild boar, hog badger	30 (23)	*Rickettisa, Coxiella,*
*Ixodes*	Hubei, Jiangxi, Jiangsu, Zhejiang, Hainan, Inner Mongolia, Jilin, Tibet, Heilongjiang	Cattle, goat, Eurasian badger, hog badger, rabbit, bat, pteromyini, tupaia	22 (16)	*Rickettisa, Coxiella, Wolbachia*
*Carios*	Hubei, Henan	–	2 (2)	*Coxiella, Rickettisa, Borrelia*
*Argas*	Heilongjiang, Xinjiang	Chicken	2 (2)	*Rickettsia, Coxiella*
Total	–	–	96 (74)	–

The number of libraries denotes that how many libraries are included in each tick genus while bracketed numbers denote the numbers of mt genomes successfully obtained in each group

### Mitochondrial genomes of 74 ticks of 46 species

We sequenced the total DNA of 96 individual or mixed tick samples, which generated an average of 7.87 Gb of clean reads for de novo assembly and annotation. Complete or near-complete mt genome sequences were successfully obtained from 74 of the 96 libraries, including 23 species whose mt genomes were reported for the first time. The length of the newly identified mt genomes ranged from 14,428 bp to 15,307 bp, and the AT content varied from 72.29% (*Ixodes* sp. A29) to 81.06% (*Hae. danieli* Z14), similar to previously identified mt genomes of ticks [9]. Furthermore, the structure, composition and arrangement of genes largely followed their closest relatives within the same genus [9, 56]. The only differences were observed in the length and composition of non-coding regions, some of which contain more than one tandem repeat region (Additional file 1: Fig. S1–S2, Table S2). For example, the mt genome of *D. marginatus* E48 has an extra copy of the non-coding region so that its length (i.e. 15,307 bp) has surpassed that of *Ixodes tasmani* (NC 041,086.1, 15,227 bp) [9] to become the longest tick mt genome identified to date (Additional file 1: Fig. S1). Furthermore, we found inconsistencies in the control region within some individual samples. For example, cloning of sequencing of PCR products spanning the control region between *trnQ* and *trnF* reveals various copy numbers of short repeat sequences within the same (single tick, *D. marginatus* E1) sample (Additional file 1: Fig. S2C).

### Molecular identification and genetic diversity of ticks

**Fig. 2:**
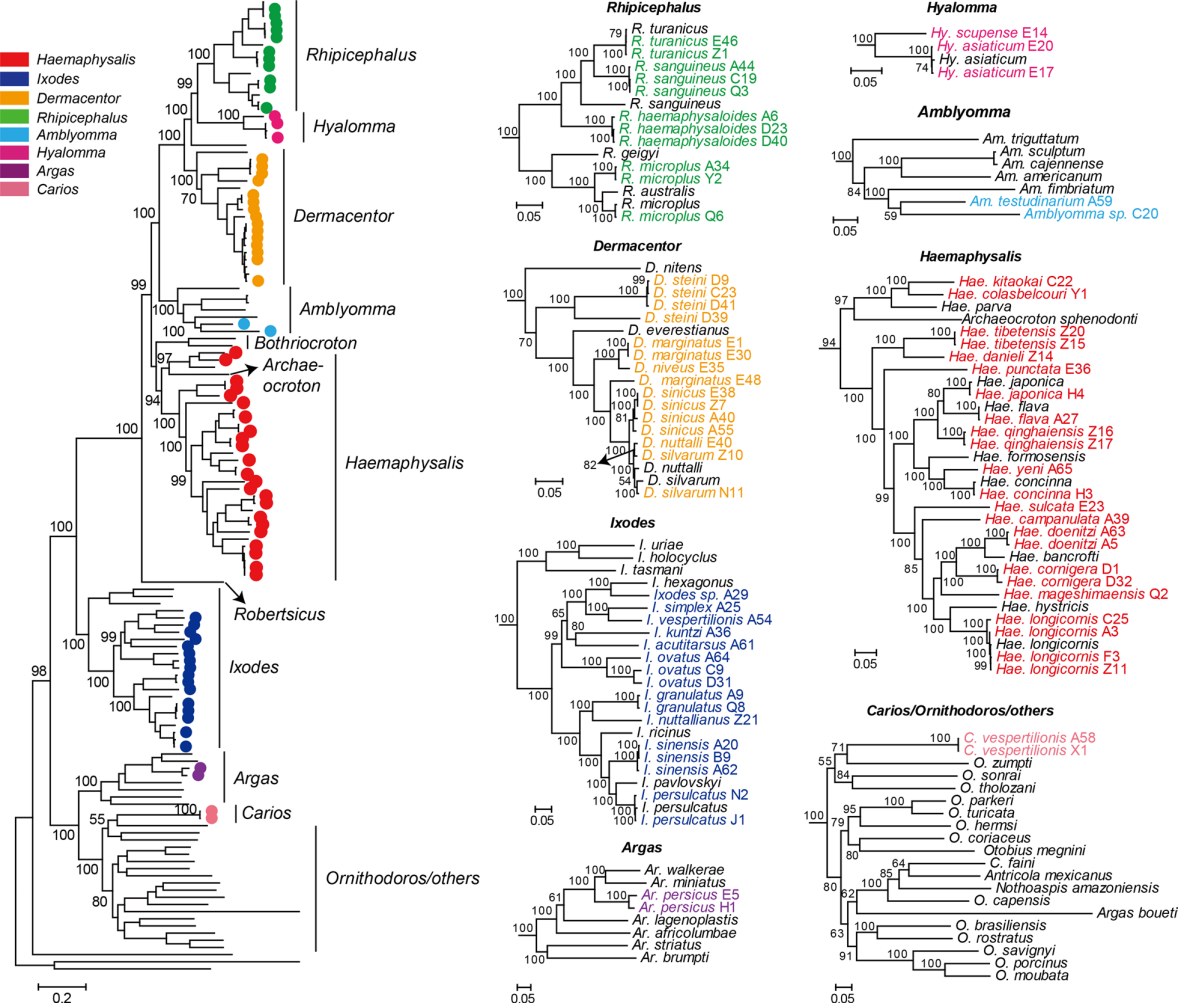
ML phylogenies of ticks based on all 13 protein coding genes and two rRNA genes. Two mite species act as the outgroup and the scale bar represents the number of nucleotide substitutions per site. For clarity, bootstrap values only shown for major nodes. The core phylogenetic tree is shown on the left, and sequences generated in this study are marked by a circle and colored according to different tick genera. The detailed subtrees of each group are shown on the right. Within each subtree, the sequences newly identified here are marked are colored accordingly along with number of sequencing libraries

Both maximum likelihood (ML) and Bayesian phylogenetic trees were estimated based on sequences of 13 protein coding genes and two rRNA genes derived from 136 mt genomes of tick, including 74 generated in this study and 62 reference mt genomes from GenBank. The ML and Bayesian methods resulted in highly similar tree topologies that placed the diversity of Chinese ticks within a global context with high resolution (Fig. 2, Additional file 1: Fig. S3–S6). Importantly, the newly added genomes greatly expanded the diversity of many groups, particularly the genera *Haemaphysalis, Ixodes* and *Dermacentor* (Fig. 2). In addition to the new species identified in this study, 23 tick species previously only known through morphological characteristics or incomplete mt genomes (e.g. *I. kuntzi*, *I. acutitarsus*, *Hae. mageshimaensis* and *Haemaphysalis colasbelcouri*) were also included (Fig. 2, Additional file 1: Fig. S3–S6). Furthermore, at least five potential cryptic species were identified—*R. sanguineus* s.l., *D. steini, D. marginatus* and *I. ovatus*—adding to the previously reported cryptic species identified in *R. microplus* [6, 57]. Each contained at least two divergent (70.93–94.21% identity) phylogenetic clusters while sharing the same morphological characteristics based on palps, basis capituli, shape and ornamentation on scutum, spurs on coxae I–IV, syncoxae and ala, etc., although more morphological features need to be examined to confirm this observation (Fig. 2). Conversely, *D. sinicus*, *Dermacentor nuttalli* and *D. silvarum* shared a very close relationship (> 98.46% identity) even though these were separate species based on morphological characteristics. Interestingly, *D. nuttalli* and *D. silvarum* cannot be distinguished based on mt genome phylogeny, although they had quite distinctive trochanter I dorsal spur (Additional file 1: Fig. S7).

### Discovery and characterization of bacterial endosymbionts and pathogens in ticks

**Fig. 3:**
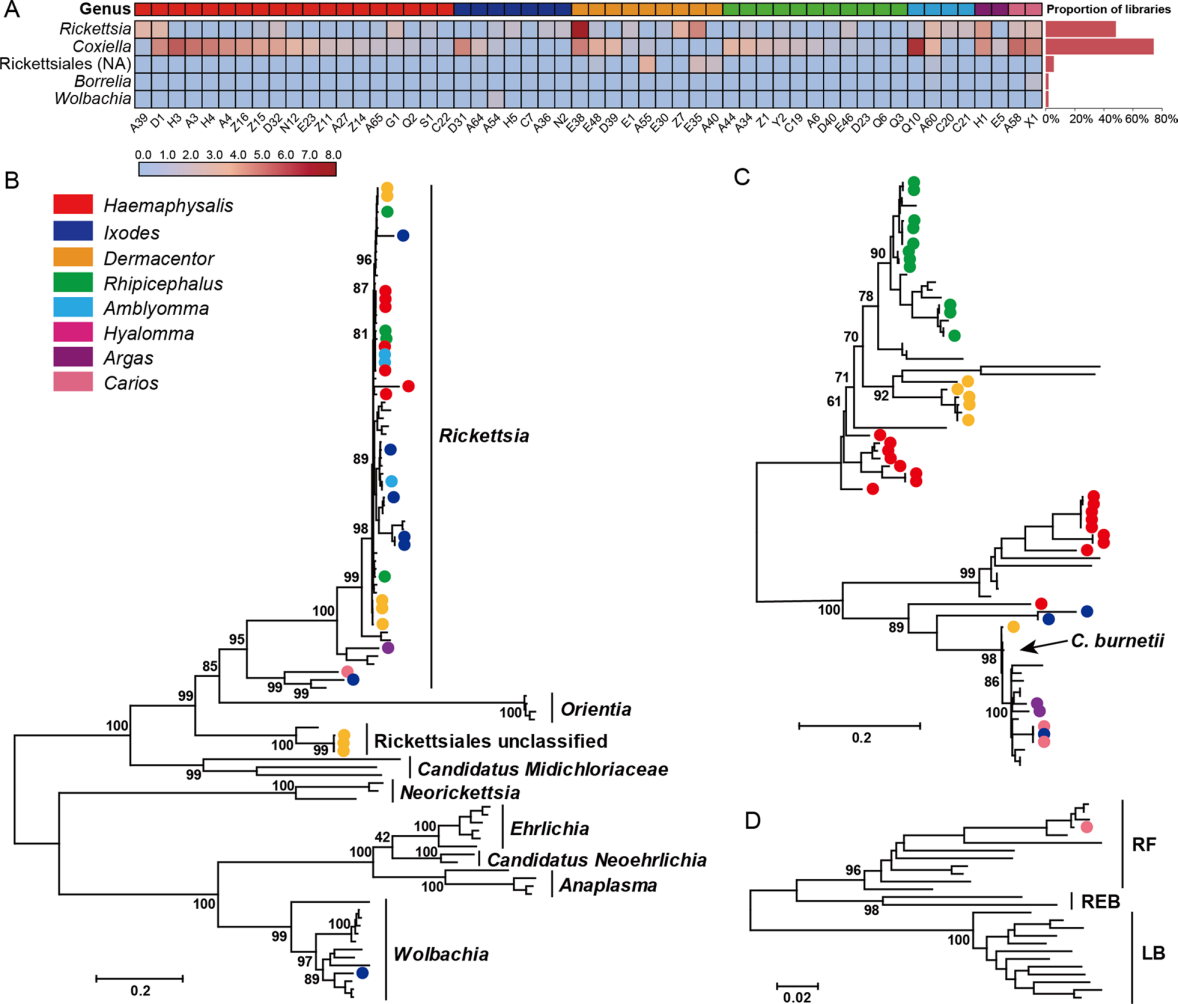
The abundance of tick-associated bacterial groups based on the *groEL* gene and the proportion of positive libraries of each group; Rickettsiales (NA) represented bacteria identified could not classified in a specific genus (**A**). Phylogenetic trees for bacteria from the order Rickettsiales based on *groEL* gene (**B**), genera *Coxiella* based on *groEL* gene (**C**) and *Borrelia* based on *flaB* gene (**D**). The trees were midpoint-rooted, and the scale bar represents the number of nucleotide substitutions per site. Sequences generated in this study marked by a circle and colored according to different tick genera. Bootstrap values only shown for major nodes. Within the Rickettsiales phylogeny, different genera are denoted by vertical lines. Within the *Coxiella* phylogeny, the position of *C. bernetii* is highlighted by a black arrow. Within the *Borrelia* phylogeny, “RF” denotes the Relapsing fever group, “REB” denotes the Reptile and echnida-associated *Borrelia*, while “LB” indicates the Lyme borreliosis group [69]

We first used metaphlan2 [40] for bacterial taxonomic profiling, which revealed the presence of > 32 genera including *Acinetobacter*, *Pseudomonas*, *Helicobacter* and *Escherichia* (Additional file 1: Fig. S8). Among these, we identified of tick endosymbiotic bacteria or bacteria that are known to harbor human pathogens: namely, the order Rickettsiales, genus *Coxiella* and genus *Borrelia*. These discoveries were further confirmed and characterized with analyzing the marker genes (Fig. 3A). Overall, 56% (54/96) of tick libraries were positive for these bacterial groups, among which *Coxiella* had the highest prevalence (40/96, 42%), followed by *Rickettsia* (26/96, 27%), *Wolbachia* (1/96, 1%) and *Borrelia* (1/96, 1%) (Fig. 3A, Additional file 1: Table S3).

Most of the species in the order Rickettsiales belonged to the genus *Rickettsia*, within which 14 bacterial species were identified from all tick genera included in this study (with the exception of *Hyalomma*; Fig. 3B), including a number of human pathogens. For example, *Rickettsia raoultii,* which causes human tick-borne lymphadenitis [58, 59], was identified from *D. marginatus* and *D. niveus* in Jinghe, Xinjiang province, a region where *R. raoultii* have previously been reported [60–62]. Within Xinjiang (Jinghe and Yining), we identified *Rickettsia sibirica* and *Rickettsia africae* circulating in *D. sinicus* and *Ixodes vespertilionis*, which are responsible for a range of tick-borne diseases, including Siberian tick typhus (STT) in Asia and African tick bite fever (ATBF) in Africa [20]. In addition, we discovered *Rickettsia heilongjiangensis*, the newly reported agent of Far-Eastern spotted fever (FESF) [63]. This bacterium was previously found in *D. silvarum* ticks from Heilongjiang, and herein it was associated with the tick species *Haemaphysalis campanulata* and *Haemaphysalis cornigera* in Hubei and Jiangxi provinces located in central China. Other pathogenic *Rickettsia* species included *Rickettsia tamurae, Rickettsia monacensis* and *Rickettsia helvetica* identified from *Amblyomma testudinarium*, *I. sinensis* and *I. kuntzi*.

We found a potentially novel species within Spotted Fever Group that was relatively divergent (< 99.46% genetic identity in six genes) from the other bacteria in this group (Additional file 1: Table S4). Since the species were identified from *Haemaphysalis megaspinosa*, we tentatively named it *Rickettsia* endosymbiont of *Haemaphysalis megaspinosa.* Furthermore, we identified four genetically divergent *Rickettsia* species that occupied basal phylogenetic positions. Among these, *Rickettsia* endosymbiont *of Ixodes persulcatus* H5 and N2 clustered with *Rickettsia canadensis* (97.78% identity), *Rickettsia* endosymbiont of *Argas persicus* H1 fell with *Rickettsia bellii* and *Rickettsia* sp. MEAM1 (*Bemisia tabaci*) (92.16% and 90.96% identity), and *Rickettsia* endosymbiont of *Ixodes vespertilionis* A54b, *Rickettsia* endosymbiont of *Carios vespertilionis* X1 formed a monophyletic group with *Rickettsia* endosymbiont of *Culicoides newsteadi* despite a high level of divergence (86.08% and 85.97% identity, respectively) (Additional file 1: Table S4, Fig. S9–S11).

In addition to *Rickettsia*, we identified two potential new species within the order Rickettsiales. One, *Wolbachia* endosymbiont of *Ixodes vespertilionis* A54, clustered with *Wolbachia pipientis* strain FL2016 (95.37%) and *Wolbahcia* endosymbiont of *Drosophila melanogaster* (95.16%) within the genus *Wolbachia*. The identification of *Wolbachia* in ticks has been reported in recent years [64, 65]. The other—*Rickettsiales* endosymbiont of *Dermacentor*—clustered with an unclassified Rickettsiales bacterium Ac37b identified from *Amblyomma cajennense* in Brazil (86.62% identity). Together they may represent a new genus or even family within the Rickettsiales (Additional file 1: Table S4, Fig. S9–S10).

Bacteria of the genus *Coxiella* had the highest prevalence among the tick species examined (40/96, 42%). The newly discovered *Coxiella* species in the present study are highly diverse and greatly expand the genetic diversity within this group (Fig. 3C). Indeed, new genetic lineages were defined based on our phylogenetic analysis, including the *Coxiella* endosymbiont *of Dermacentor marginatus*, the *Coxiella* endosymbiont *of Haemaphysalis concinna* and the *Coxiella* endosymbiont *of Ixodes ovatus*, most of which were divergent from existing members of *Coxiella* and generally associated with specific tick genera (Fig. 3C, Additional file 1: Fig. S12). *Coxiella burnetii*, the causative agent of Q fever [66], has been reported from *D. sinicus* tick sampled from Xinjiang province and was closely related with the “Dugway 5J108-111” strain sampled from the US [67] (Fig. 3C, Additional file 1: Fig. S11). In addition to *C. burnetii*, we identified a single species of *Borrelia* from a soft tick *Carios vesperitilionis* in Henan province. Based on the phylogenetic analyses, the newly identified bacteria, named *Borrelia henanensis* X1, fell within a clade “RF” that contains pathogens causing tick-borne relapsing fever (Fig. 3D, Additional file 1: Fig. S13) [68, 69].

### Ecological and evolutionary patterns in ticks and their associated bacterial symbionts

We used Mantel tests to examine whether the tick host and/or geographic factors shape the genetic diversity of the bacteria they carry. For both *Rickettsia* and *Coxiella*, our results revealed positive and significant (*P* < 0.0005) correlations between tick and bacteria genetic distance matrices. However, no such significant correlation was found between bacterial genetic distance and geographic distance. Similar results were obtained using (i) partial Mantel analyses, in which we tested the effect between two factors while controlling for the third, and (ii) multiple linear regression analyses in which we tested the effect between three matrices (Table 2, Additional file 1: Table S5). These results suggested that bacterial genetic diversity was primary shaped by tick genetic distance, with geographic distribution having little or no impact. The strong impact of tick on bacterial genetic diversity was also reflected in the phylogenetic analysis in which we observed a significant clustering of bacterial genetic diversity at the tick general level [*Rickettsia*: association index (AI) = 2.760, *P* < 0.001; *Coxiella*: AI = 0.969, *P* < 0.001].

**Table 2:** Results of the Mantel test and partial Mantel test comparing two factors (tick genetic distance and geographic distance) that predict the structure of genetic diversity in bacterial pathogens

	Model	*r* value (*P* value)
*Rickettsia*	Tick^a^	0.5868 (0.0004)
	Tick^a^ | geography^b^	0.5952 (0.0009)
	Geography^a^	0.0028 (0.3916)
	Geography | tick^b^	− 0.1225 (0.8633)
*Coxiella*	Tick^a^	0.4939 (0.0001)
	Tick^a^ | geography^b^	0.4939 (0.0001)
	Geography^a^	0.0013 (0.4499)
	Geography | tick^b^	0.0053 (0.4378)

^a^Mantel test

^b^Partial Mantel test

| Indicates that the first factor excludes the effect of the second

**Fig. 4:**
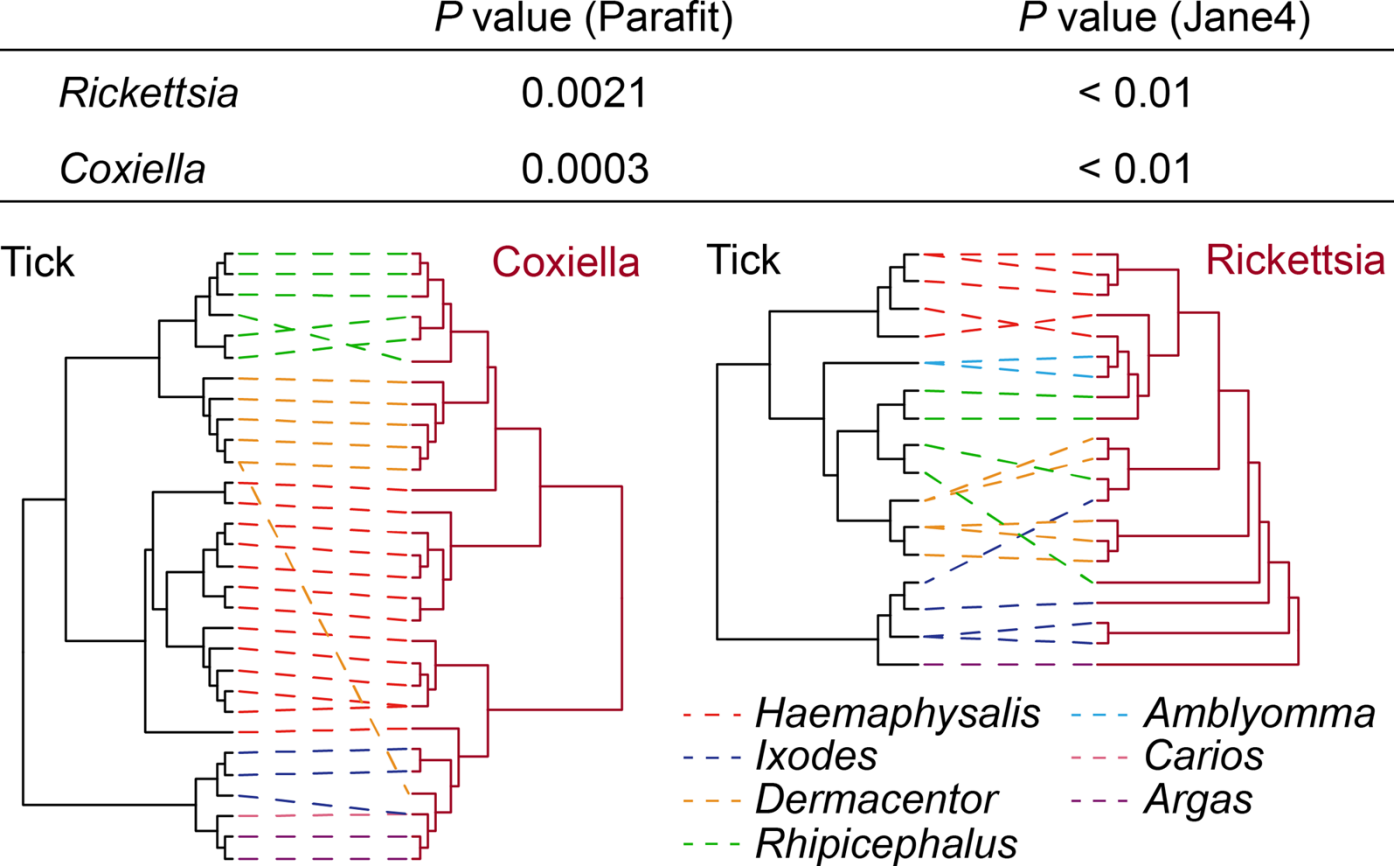
Co-phylogenetic comparisons of *Rickettsia* and *Coxiella* bacteria phylogenies and their corresponding tick hosts. The table shows the results of the co-phylogeny analysis using Parafit and Jane4. The tanglegram shows the match between the phylogenies of the bacteria and tick hosts. The relationship between the two phylogenies is displayed to maximize topological congruence. Dotted line colors correspond to different tick groups as shown by figure legend at the right bottom

We next examined whether the phylogeny of the ticks and their bacterial symbionts exhibited a pattern of bacterial-tick co-divergence over evolutionary time. We first tested hypothesis of co-divergence using an event-based framework, based on which we reconciled the phylogenies of ticks and their associated bacteria (i.e. *Rickettsia* and *Coxiella*, respectively) by accounting for four processes: co-divergence, duplication, host switching and loss [43]. This revealed significantly fewer non-co-divergence events (i.e. duplication, host switching and loss) than expected by chance alone. We similarly examined the co-divergence hypothesis using a distance method, in which we evaluated the overall phylogenetic congruence by comparing the tick and bacterial symbionts patristic distance [44]. This confirmed the significant overall similarity (ParafitGlobal, *P* = 0.0021 and 0.0003, respectively, for *Rickettsia* and *Coxiella*, at 9999 permutations) between the tick and bacterial symbionts phylogenies (Fig. 4). Collectively, these results suggest that the symbiotic bacteria from genera *Rickettsia* and *Coxiella* have co-diverged with their tick hosts for at least 264 million years.

## Discussion

We collected > 20,000 ticks and determined the mitochondrial genomes of at least 46 species representing the diversity of both common and rare ticks in China. Our sampling mostly covered human residential areas as well as some biodiversity hotspots, namely Shennongjia forest (Hubei province), the Tibet plateau (Tibet and Qinghai provinces) and Hulun Beir prairie (Inner Mongolia). While most ticks were sampled from domestic animals and were commonplace [6], those identified from wildlife or directly from the environment yielded more unique diversity. Hence, there may be many more species of ticks in China that have not yet been identified by current disease or human-centered sampling schemes. Indeed, a number of tick species, such as *I. kuntzi* and *Hae. colasbelcouri* identified from Taiwan and Laos [70, 71], have not been sequenced and characterized previously. Furthermore, there is a general lack of genomic surveillance of ticks in reptiles and amphibians, such that substantial evolutionary gaps remain in studying the long-term tick-bacteria relationship.

Our study inferred the evolutionary history of ticks based on the entire sets of mitochondrial genes, which revealed a well-supported phylogenetic tree that resolves the inter-species relationships of ticks with high resolution (Fig. 2). Mitochondrial genes are an important tool in tick molecular systematics because they evolve faster than most nuclear genes and are therefore better suited to address evolutionary questions at lower taxonomic levels [8, 53, 54], and their small size and the abundance of mitochondria in cells make them easy to analyze. Indeed, the sequencing depth in this study (0.2–30.1 Gbp per library) is sufficient for mitochondrial genes but inadequate for nuclear genes.

The results of the morphological and molecular species identification performed here were largely similar, suggesting that sequencing can reliably identify tick species. However, there were several inconsistencies, mainly reflected in the presence of five cryptic species complexes, within which morphologically identical ticks can be separated by high levels of intra-specific genetic diversity (e.g. 73.40%, 70.93%) or had paraphyletic phylogenetic structure (e.g. *D. marginatus* and *R. sanguineus* s.l.) (Fig. 2). Although we treat all these as cryptic species, we could not completely exclude that there may be some additional morphological characteristics that can distinguish these ticks and were unrecognized during our species identification process. In addition to cryptic species, inconsistencies between morphological and mitochondrial taxonomy were also reflected in the observation that several morphologically distinct ticks (*D. nuttali*, *D. silvarum*, *D. sinicus*) had very similar mt sequences (i.e. > 98.46% genetic identity). This suggests a relatively recent speciation event, although this needs to be further confirmed with nuclear genes.

Our study also revealed the high prevalence and large diversity of bacterial endosymbionts within the ticks examined, many of which fell with groups/species that contain human pathogens, including members of Spotted Fever Group (nine species, genus *Rickettsia*), *C. burnetii* as well as *B. henanensis*. Given that our study only covers 219 individuals from 46 species of ticks, the prevalence level for pathogenetic bacteria within these ticks is relatively high. This broadly agrees with previous studies that revealed a 41.2%—68.5% prevalence for pathogenetic bacteria [72, 73]. Furthermore, the abundance of some of the bacterial pathogens was high (1.42—162.57 RPM), further facilitating inter-tick transmission. Interestingly, we did not detect other pathogenic genera within order Rickettsiales that are frequently carried by ticks, such as *Anaplasma* and *Ehrlichia*, although this may simply reflect a limited sample size.

Our results greatly enrich the diversity of both ticks and their associated bacteria, revealing that both endosymbiotic bacteria groups, namely those of the *Rickettsia* and *Coxiella* genera, had close association with ticks. This is also reflected in both the strong tick structure on the bacteria phylogeny and the high resemblance between bacteria and tick phylogenies indicative of long-term co-divergence. Previous studies have suggested a lack of co-divergence between *Rickettsia* [74] and *Coxiella*-like endosymbionts [27]. However, these were mainly based on bacteria detected from PCR assays that are highly sensitive and may easily include both endosymbionts as well as bacteria transmitted through co-feeding. In addition, some previous studies of co-divergence are based on the rRNA gene, which is too conserved to distinguish closely related species [75]. In contrast, the unbiased metagenomic sequencing performed here only detects those bacteria at relatively high abundance, such that these data are more reflective of the presence of endosymbiotic bacteria. Clearly, more data are required to fully resolve the co-evolutionary history between ticks and their endosymbiont bacteria.

## Conclusions

In sum, our analysis of > 20,000 ticks collected over broad geographic range across China provides insights into diversity and evolutionary history of ticks and their associated bacteria symbionts/pathogens. Our data reveal that the genetic diversity of ticks in China goes beyond a few common species and includes rare and under-explored species, for which more diversity in wildlife hosts that remains to be discovered. Importantly, despite their low occurrence, these uncommon tick species can harbor diverse pathogens, some of which could pose a potential threat to human health.

## Supplementary Information

**Additional file 1: Table S1.** Detailed information on the 96 tick libraries examined here. **Figure S1.** Gene arrangements in tick mitochondrial genomes across eight genera. There are 23 linear maps in 19 species of *Haemaphysalis*, nine in *Amblyomma* species, two in *Bothriocroton* species, three in two *Hyalomma* species, 13 in 10 *Rhipicephalus* species, 18 in 16 Ixodes species, one of Nuttalliellidae species and 26 linear maps of Argasid ticks. Each mitochondrial genome has 13 protein-coding genes, two ribosomal genes, 22 tRNA genes and misc-features varying with tick species. Mitochondrial gene arrangement and direction in *Haemaphysalis*, *Dermacentor*, *Amblyomma*, *Hyalomma*, *Rhipicephalus* and *Bothriocroton* are almost identical and those in Ixodes, Nuttalliellidae and Argasidae are almost identical. Protein-coding genes are denoted by yellow arrows, rRNA are denoted by red arrows, tRNA are denoted by pink arrows, and control regions are denoted by gray arrows. The direction of arrows indicated the direction of protein translation. Abbreviations are as follows: ND1 = NADH dehydrogenase subunit 1, ND2 = NADH dehydrogenase subunit 2, ND3 = NADH dehydrogenase subunit 3, ND4 = NADH dehydrogenase subunit 4, ND4L = NADH dehydrogenase subunit 4L, ND5 = NADH dehydrogenase subunit 5, ND6 = NADH dehydrogenase subunit 6, COX1 = cytochrome oxidase I, COX2 = cytochrome oxidase II, COX3 = cytochrome oxidase III, ATP8 = ATPase8, ATP6 = ATPase8, 16 s rRNA = large ribosomal subunit, 12 s rRNA = small ribosomal subunit, CYTB = cytochrome b. **Figure S2.** Sequence alignment of special tandem repeat regions in misc-features of *Dermacentor* mt genome. The first hypervariable region located between tRNA-Gln and NAD1 (A), and the second hypervariable region located tRNA-Gln between tRNA-Phe (B), and its cloning of sequencing of PCR products spanning the control region implied various copy numbers of short repeat sequences within the same *D. marginatus* E1 sample (C). **Table S2.** Special tandem repeat regions in misc-features of *Dermacentor* mt genome. **Figure S3.** Detailed phylogenetic tree of ticks based on all 13 protein-coding genes and two rRNA genes inferred using both ML and Bayesian methods with two mites as the outgroup. Highly similar tree topologies were obtained. The trees are midpoint-rooted, and the scale bar represents the number of nucleotide substitutions per site. For clarity, the bootstrap values are shown on the left with posterior probability on the right for each node. Mt genomes identified in this study are marked colored font according to different tick genera; the name of each tick genus is shown beside. **Figure S4.** Detailed phylogenetic tree of ticks based on only 13 protein-coding genes estimated using both ML and Bayesian methods with two mites as the outgroup. Highly similar tree topologies were obtained. The trees are midpoint-rooted, and the scale bar represents the number of nucleotide substitutions per site. For each node, the bootstrap values are shown on the left with posterior probability on the right. Mt genomes identified in this study are marked colored font according to different tick genera; the name of each tick genus is shown beside. **Figure S5.** Detailed phylogenetic tree of ticks based on mt 12S rRNA gene estimated using ML methods. The tree is midpoint-rooted, and the scale bar represents the number of nucleotide substitutions per site. Mt 12S rRNA gene identified in this study are marked colored font according to different tick genera, the name of each tick genus is shown beside. **Figure S6.** Detailed phylogenetic tree of ticks based on mt 16S rRNA gene estimated using ML methods. The tree is midpoint-rooted, and the scale bar represents the number of nucleotide substitutions per site. Mt 16S rRNA gene identified in this study are marked colored font according to different tick genera, the name of each tick genus is shown beside. **Figure S7.** Differential diagnosis for *D.* *nuttalli* and *D.* *silvarum*. Adult trochanter l dorsal spur of *D.* *nuttalli* (A) is short, broad and blunt apically, while that of *D.* *silvarum* (B) is slightly long, pointed at the apex. **Figure S8.** Relative abundance of bacteria and fungi at the level of genus based on metaphlan2 results. **Table S3.** Prevalence of tick-borne bacteria in ticks at the level of genus. A total of 43 tick associated bacterial species were identified from 54 libraries and 7 tick genera (with the exception of *Hyalomma*). Bracketed numbers denote numbers of bacteria identified in each bacterial group at the level of genera. **Table S4.** The genetic similarity of bacterial strains identified in this study with its closest reference sequence. **Figure S9.** ML phylogenetic tree of the order Rickettsiales based on the groEL gene. The tree is midpoint-rooted, and the scale bar represents the number of nucleotide substitutions per site. The strains identified here are marked by colored font according to different tick genera, and reference sequences are represented by black font with corresponding accession number nearby. Each bacterial group at genus level is denoted by gray font and a vertical line, while bacterial group at subfamily level is denoted by black font and a square bracket. **Figure S10.** ML phylogenetic tree of the order Rickettsiales based on the gltA gene. The tree is midpoint-rooted, and the scale bar represents the number of nucleotide substitutions per site. The strains identified here are marked by colored font according to different tick genera, and reference sequences are represented by black font with corresponding accession number nearby. Each bacterial group at genus level is denoted by gray font and vertical line, while bacterial group at subfamily level is denoted by black font and square bracket. **Figure S11.** ML phylogenetic tree of the genus *Rickettsia* based on the six conserved housekeeping genes, including the atpD, coxB, ftsZ, gltA, groEL and sucA genes. Individual genes were first aligned and then concatenated to form super-alignment for phylogenetic analysis. The tree is midpoint-rooted, and the scale bar represents the number of nucleotide substitutions per site. The strains identified here are marked by colored font according to different tick genera, and reference sequences are represented by black font with corresponding accession number nearby. The Rickettsiae collected here could further assigned into three groups: Spotted fever group (SFG), Typhus group (TG) and Belli group (BG). **Figure S12.** ML phylogenetic tree of the genus *Coxiella* based on the groEL gene. The tree is midpoint-rooted, and the scale bar represents the number of nucleotide substitutions per site. The strains identified here are marked by colored font according to different tick genera, and reference sequences are represented by black font with corresponding accession number nearby. A significant clustering of *Coxiella* genetic diversity at the host general level was observed, and the position of *C. burnetii* was highlighted by an arrow. **Figure S13.** ML phylogenetic tree of the genus *Borrelia* based on the flaB gene. The tree is midpoint-rooted, and the scale bar represents the number of nucleotide substitutions per site. The trees are midpoint-rooted, and the scale bar represents the number of nucleotide substitutions per site. The *B.* *henanensis* strain X1 identified here is marked in pink font according to its tick genus (*Carios*), and reference sequences are represented by black font with corresponding accession number nearby. The *Borrelia* collected here could be assigned into three groups: The Relapsing fever group (RFG), Reptile and echidna-associated *Borrelia* (REB) and Lyme borreliosis group (LB). **Table S5.** Multiple regression of bacteria genetic distance against tick genetic distance and geographic distance shows that bacterial genetic diversity was mainly structured by tick genetic distance rather than geographical distribution.

## Data Availability

The tick mt genome sequences reported in this study are available on NCBI, under accession number OM368288-OM368330, MK344649. Sequence alignments underlying analyses, phylogenetic trees and related code are available from figshare (https://figshare.com/articles/dataset/The_diversity_and_evolutionary_relationships_of_ticks_and_tick-borne_bacteria_in_China/19354481). Raw sequencing data are available at the NCBI SRA database as BioProject PRJNA816261 (https://www.ncbi.nlm.nih.gov/bioproject/PRJNA816261).

## Abbreviations

Mt: Mitochondrial
N(A)D1: NADH dehydrogenase subunit 1
N(A)D2: NADH dehydrogenase subunit 2
N(A)D3: NADH dehydrogenase subunit 3
N(A)D4: NADH dehydrogenase subunit 4
N(A)D4L: NADH dehydrogenase subunit 4L
N(A)D5: NADH dehydrogenase subunit 5
N(A)D6: NADH dehydrogenase subunit 6
COX1: Cytochrome oxidase I
COX2: Cytochrome oxidase II
COX3: Cytochrome oxidase III
ATP8: ATPase8
ATP6: ATPase8
16 s rRNA: Large ribosomal subunit
12 s rRNA: Small ribosomal subunit
CYTB: Cytochrome b
trnL1: TRNA-Leu1
trnL_2_: TRNA-Leu2
trnC: TRNA-Cys
trnQ: TRNA-Gln
trnF: TRNA-Phe
groEL: Chaperonin GroEL
gltA: Citrate synthase
atpD: ATP synthase CF1 delta chain
coxB: Cytochrome c oxidase subunit II
ftsZ: Cell division protein FtsZ
sucA: 2-Oxoglutarate dehydrogenase E1 component
bamA: Outer membrane protein assembly factor BamA
flab: Flagellin FlaB
rpoB: RNA polymerase beta subunit
SFG: Spotted fever group
LB: Lyme borreliosis
STT: Siberian tick typhus
ATBF: Asia and African tick bite fever
FESF: Far-Eastern spotted fever
RF: Relapsing fever
ML: Maximum likelihood
BaTS: Bayesian tip-association significance testing
RPM: Reads per million total reads
AI: Association index
PBS: Phosphate buffered solution
