# Supplementary material for: The diversity and evolutionary relationships of ticks and tick-borne bacteria collected in China
Source: Parasit Vectors. 2022 Oct 1;15:352. doi: 10.1186/s13071-022-05485-3 (PMC9526939; doi:10.1186/s13071-022-05485-3)
Supplement: Supplementary file 1 — Additional file 1: Table S1. Detailed information on the 96 tick libraries examined here. Figure S1. Gene arrangements in tick mitochondrial genomes across eight genera. There are 23 linear maps in 19 species of Haemaphysalis, nine in Amblyomma species, two in Bothriocroton species, three in two Hyalomma species, 13 in 10 Rhipicephalus species, 18 in 16 Ixodes species, one of Nuttalliellidae species and 26 linear maps of Argasid ticks. Each mitochondrial genome has 13 protein-coding genes, two ribosomal genes, 22 tRNA genes and misc-features varying with tick species. Mitochondrial gene arrangement and direction in Haemaphysalis, Dermacentor, Amblyomma, Hyalomma, Rhipicephalus and Bothriocroton are almost identical and those in Ixodes, Nuttalliellidae and Argasidae are almost identical. Protein-coding genes are denoted by yellow arrows, rRNA are denoted by red arrows, tRNA are denoted by pink arrows, and control regions are denoted by gray arrows. The direction of arrows indicated the direction of protein translation. Abbreviations are as follows: ND1 = NADH dehydrogenase subunit 1, ND2 = NADH dehydrogenase subunit 2, ND3 = NADH dehydrogenase subunit 3, ND4 = NADH dehydrogenase subunit 4, ND4L = NADH dehydrogenase subunit 4L, ND5 = NADH dehydrogenase subunit 5, ND6 = NADH dehydrogenase subunit 6, COX1 = cytochrome oxidase I, COX2 = cytochrome oxidase II, COX3 = cytochrome oxidase III, ATP8 = ATPase8, ATP6 = ATPase8, 16 s rRNA = large ribosomal subunit, 12 s rRNA = small ribosomal subunit, CYTB = cytochrome b. Figure S2. Sequence alignment of special tandem repeat regions in misc-features of Dermacentor mt genome. The first hypervariable region located between tRNA-Gln and NAD1 (A), and the second hypervariable region located tRNA-Gln between tRNA-Phe (B), and its cloning of sequencing of PCR products spanning the control region implied various copy numbers of short repeat sequences within the same D. marginatus E1 sample (C). Table S2. Special tandem repeat regions [file 13071_2022_5485_MOESM1_ESM.pdf]

Additional file 1: Table S1. Detailed information on the 96 tick libraries examined here.

Strain	Tick species	Host or habitat of sampling	Place of sampling	Sex and numbers	preservation condition	Status of mt genome	Length of genome	GenBank Accession
A9	*Ixodes granulatus*	Rabbit	Daye	1 female	Cryopreservation	Near Complete	14538	OM368258
A20	*Ixodes sinensis*	Eurasian badger	Yicheng	1 female	Cryopreservation	Complete	14534	OM368259
A25	*Ixodes simplex*	Bat	Jingmen	1 female	Preserved in ethanol for 5 years	Complete	14556	OM368260
A29	*Ixodes* sp.	Hog-badger	Yichang	1 female	Cryopreservation	Complete	14545	OM368261
A36	*Ixodes kuntzi*	Pteromyini	Yichang	1 female	Cryopreservation	Complete	14524	OM368262
A41	*Ixodes ovatus*	Hog-badger	Wuhan	1 female	Preserved in ethanol for 6 years	Small fragments	NA	NA
A47	*Ixodes acutitarsus*	Hog-badger	Yichang	1 female	Cryopreservation	Small fragments	NA	NA
A54	*Ixodes vespertilionis*	Bat	Huangshi	1 nymph, 6 larvae	Preserved in ethanol for 6 years	Complete	14547	OM368263
A61	*Ixodes acutitarsus*	Hog-badger	Yichang	1 female	Cryopreservation	Complete	14481	OM368264
A62	*Ixodes sinensis*	Cattle	Wuhan	1 female	Cryopreservation	Complete	14669	OM368265
A64	*Ixodes ovatus*	Hog-badger	Wuhan	1 female	Cryopreservation	Complete	14512	OM368266
A68	*Ixodes ovatus*	Hog-badger	Wuhan	2 females, 4 males	Preserved in ethanol for 6 years	NA	NA	NA
B9	*Ixodes sinensis*	NA	Yancheng	1 female	Cryopreservation	Complete	14530	OM368267
C7	*Ixodes sinensis*	Goat	Wenzhou	1 female	Cryopreservation	NA	NA	NA
C8	*Ixodes ovatus*	Goat	Wenzhou	1 female	Cryopreservation	NA	NA	NA
C9	*Ixodes ovatus*	Goat	Wenzhou	1 female	Cryopreservation	Complete	14507	OM368268
D31	*Ixodes ovatus*	Goat	Ganzhou	1 female	Cryopreservation	Complete	14510	OM368269
H5	*Ixodes persulcatus*	Drag-flagging method	Suifenhe	2 female	Cryopreservation	Complete	14544	NA
J1	*Ixodes persulcatus*	NA	Yanbian	8 female, 1 male	Preserved in ethanol for 60 years	Near Complete	14551	OM368270
N2	*Ixodes persulcatus*	Cattle	Yakeshi	1 female	Cryopreservation	Complete	14545	OM368271
Q8	*Ixodes granulatus*	Tupaia	Chengmai	1 female	Cryopreservation	Complete	14541	OM368272
Z21	*Ixodes nuttallianus*	Drag-flagging method	Nyalam	1 female	Preserved in ethanol for 26 years	Complete	14745	OM368273
A3	*Haemaphysalis longicornis*	Drag-flagging method	Macheng	12 females	Cryopreservation	Complete	14693	OM368274
A4	*Haemaphysalis longicornis*	Drag-flagging method	Macheng	12 females	Cryopreservation	NA	NA	NA
A5	*Haemaphysalis doenitzi*	Pheasant	Wuhan	1 female	Preserved in ethanol for 6 years	Near Complete	14671	OM368275
A27	*Haemaphysalis flava*	Hedgehog	Suizhou	1 female	Cryopreservation	Complete	14685	OM368276
A39	*Haemaphysalis campanulata*	Dog	Wuhan	1 female	Cryopreservation	Complete	14691	OM368277
A63	*Haemaphysalis doenitzi*	Pheasant	Wuhan	3 males	Cryopreservation	Near Complete	14671	OM368278
A65	*Haemaphysalis yeni*	Muntjac	Wuhan	1 female	Cryopreservation	Complete	14690	OM368279
C22	*Haemaphysalis kitaokai*	Goat	Wenzhou	1 male	Preserved in ethanol for 1 year	Complete	14937	OM368280
C25	*Haemaphysalis longicornis*	Wild boar	Jinhua	1 female	Preserved in ethanol for 12 years	Complete	14695	OM368281
D1	*Haemaphysalis cornigera*	Cattle	Ganzhou	6 females	Preserved in ethanol for 1 year	Complete	14681	OM368282
D32	*Haemaphysalis cornigera*	Cattle	Ganzhou	8 females	Preserved in ethanol for 3 years	Complete	14680	OM368283
E23	*Haemaphysalis sulcata*	NA	Jinghe	1 female	Cryopreservation	Complete	14679	OM368284
E26	*Haemaphysalis punctata*	Goat	Bole	1 female	Cryopreservation	NA	NA	NA
E36	*Haemaphysalis punctata*	Cattle	Jinghe	2 females, 2 males	Preserved in ethanol for 6 years	Complete	14697	OM368285
N12	*Haemaphysalis concinna*	Cattle	Yakeshi	2 females, 6 males	Cryopreservation	Complete	14678	NA
F3	*Haemaphysalis longicornis*	Cattle	Shenyang	1 female	Cryopreservation	Complete	14694	OM368286
G1	*Haemaphysalis yeni*	NA	Wuyishan	1 female, 1 male	Preserved in ethanol for 60 years	2 Complete		NA
H3	*Haemaphysalis concinna*	Drag-flagging method	Suifenhe	2 females	Cryopreservation	Complete	14677	OM368287
H4	*Haemaphysalis japonica*	Drag-flagging method	Suifenhe	4 females	Cryopreservation	Complete	14677	OM368288
Q2	*Haemaphysalis mageshimaensis*	Dog	Haikou	1 female	Cryopreservation	Complete	14721	OM368289
Q11	*Haemaphysalis lagrangei*	Dog	Haikou	1 female, 1 male	Preserved in ethanol for 60 years	NA	NA	NA
S1	*Haemaphysalis megaspinosa*	Hog-badger	Hanzhong	1 male	Preserved in ethanol for 20 years	Partial	NA	NA
Y1	*Haemaphysalis colasbelcouri*	Cattle	Qujing	1 female	Cryopreservation	Complete	14885	OM368290
Z11	*Haemaphysalis longicornis*	Drag-flagging method	Beijing	1 female, 1 male, 8 larvae	Cryopreservation	Complete	14694	OM368291
Z13	*Haemaphysalis qinghaiensis*	Yak	Haidong	1 female	Cryopreservation	NA	NA	NA
Z14	*Haemaphysalis danieli*	Feeding	Haidong	2 females, 2 males	Cryopreservation	Complete	14739	OM368292
Z15	*Haemaphysalis tibetensis*	Feeding	Lhasa	1 female	Cryopreservation	Complete	14739	OM368293
Z16	*Haemaphysalis qinghaiensis*	Yak	Haidong	1 female	Cryopreservation	Complete	14683	OM368294
Z17	*Haemaphysalis qinghaiensis*	Yak	Haidong	2 males	Cryopreservation	Complete	14678	OM368295
Z20	*Haemaphysalis tibetensis*	Feeding	Lhasa	1 female, 3 nymphs	Cryopreservation	Complete	14725	OM368296
A6	*Rhipicephalus haemaphysaloides*	Cattle	Huangshi	1 male	Cryopreservation	Complete	14742	MK344649
A34	*Rhipicephalus microplus*	Cattle	Jingmen	1 female	Cryopreservation	Complete	14900	OM368321

A44	*Rhipicephalus sanguineus*	Dog	Wuhan	8 males	Preserved in ethanol for 6 years	Complete	14713	OM368322
C19	*Rhipicephalus sanguineus*	Dog	Ningpo	1 female	Cryopreservation	Complete	14714	OM368323
D23	*Rhipicephalus haemaphysaloides*	Cattle	Ganzhou	1 female, 1 male	Preserved in ethanol for 1 year	Complete	14744	OM368324
D34	*Rhipicephalus haemaphysaloides*	Drag-flagging method	Yingtan	1 female, 1 male	Cryopreservation	NA	NA	NA
D40	*Rhipicephalus haemaphysaloides*	Drag-flagging method	Yingtan	1 female	Cryopreservation	Near Complete	14743	OM368325
E46	*Rhipicephalus turanicus*	Dog	Yining	1 male	Cryopreservation	Complete	14719	OM368326
Q3	*Rhipicephalus sanguineus*	Dog	Wuzhishan	1 male	Cryopreservation	Complete	14711	OM368327
Q6	*Rhipicephalus microplus*	Cattle	Haikou	5 males	Cryopreservation	Complete	14899	OM368328
Y2	*Rhipicephalus microplus*	Cattle	Qujing	1 female	Cryopreservation	Complete	14863	OM368329
Z1	*Rhipicephalus turanicus*	Hedgehog	Beijing	1 female	Cryopreservation	Complete	14717	OM368330
A40	*Dermacentor sinicus*	Rabbit	Wuhan	6 males	Cryopreservation	Complete	14996	OM368297
A55	*Dermacentor sinicus*	Rabbit	Jingmen	6 males	Preserved in ethanol for 5 years	Complete	14996	OM368298
C23	*Dermacentor steini*	Wild boar	Jinhua	2 males	Preserved in ethanol for 12 years	Complete	14780	OM368299
C24	*Dermacentor steini*	Wild boar	Jinhua	1 female	Preserved in ethanol for 12 years	Near Complete	14775	NA
D33	*Dermacentor steini*	Drag-flagging method	Shangrao	1 male	Cryopreservation	NA	NA	NA
D9	*Dermacentor steini*	Goat	Nanchang	2 nymphs	Preserved in ethanol for 3 years	Complete	14785	OM368300
D39	*Dermacentor steini*	Drag-flagging method	Yingtan	1 female	Preserved in ethanol for 3 years	Complete	14796	OM368301
D41	*Dermacentor steini*	Drag-flagging method	Yingtan	1 female	Preserved in ethanol for 1 year	Complete	14767	OM368302
E1	*Dermacentor marginatus*	NA	Jinghe	1 male	Preserved in ethanol for 3 years	Near Complete	15178	OM368303
E30	*Dermacentor marginatus*	Cattle	Jinghe	1 male	Preserved in ethanol for 6 years	Complete	15093	OM368304
E34	*Dermacentor niveus*	Cattle	Jinghe	1 male	Preserved in ethanol for 6 years	Near Complete	15121	NA
E35	*Dermacentor niveus*	Cattle	Jinghe	1 male	Preserved in ethanol for 6 years	Complete	15110	OM368305
E38	*Dermacentor sinicus*	Cattle	Jinghe	1 female	Preserved in ethanol for 6 years	Complete	14991	OM368306
E40	*Dermacentor nuttalli*	Cattle	Jinghe	1 male	Preserved in ethanol for 40 years	Complete	15086	OM368307
E48	*Dermacentor marginatus*	NA	Zhaosu	1 female	Preserved in ethanol for 60 years	Near Complete	15299	OM368308
N11	*Dermacentor silvarum*	Cattle	Yakeshi	1 male	Cryopreservation	Complete	15086	OM368309
Z10	*Dermacentor silvarum*	Feeding	Zhangjiakou	1 male	Cryopreservation	Complete	14950	OM368310
Z7	*Dermacentor sinicus*	Hedgehog	Beijing	2 males	Cryopreservation	Complete	14947	OM368311
A74	*Hyalomma scupense*	Cattle	Shiyan	1 female	Preserved in ethanol for 60 years	Several gaps	NA	NA
E14	*Hyalomma scupense*	Cattle	Jinghe	1 male	Cryopreservation	Complete	14721	OM368314
E17	*Hyalomma asiaticum*	Goat	Jinghe	1 male	Cryopreservation	Complete	14723	OM368315
E20	*Hyalomma asiaticum*	Camel	Tacheng	1 male	Cryopreservation	Complete	14723	OM368316
A59	*Amblyomma testudinarium*	Wild boar	Wuhan	1 male	Preserved in ethanol for 6 years	Complete	14976	OM368312
A60	*Amblyomma testudinarium*	Wild boar	Wuhan	2 males	Preserved in ethanol for 6 years	NA	NA	NA
C20	*Amblyomma* sp.	Pangolin	Jinhua	1 male	Cryopreservation	Complete	14760	OM368313
C21	*Amblyomma* sp.	Pangolin	Jinhua	2 males	Cryopreservation	NA	NA	NA
Q9	*Amblyomma javanense*	Pangolin	Haikou	1 male	Preserved in ethanol for 60 years	Partial	NA	NA
Q10	*Amblyomma javanense*	Pangolin	Haikou	1 female	Preserved in ethanol for 60 years	NA	NA	NA
E5	*Argas persicus*	Chicken coop	Tacheng	1 female	Cryopreservation	Complete	14428	OM368319
H1	*Argas persicus*	Cattle shed	Hegang	1 female	Preserved in ethanol half year	Complete	14432	OM368320
A58	*Carios vespertilionis*	Hotel	Zhongxiang	1 male	Preserved in ethanol for 6 years	Complete	14528	OM368317
X1	*Carios vespertilionis*	Hotel	Xuchang	4 females, 4 males	Preserved in ethanol for 3 years	Complete	14529	OM368318

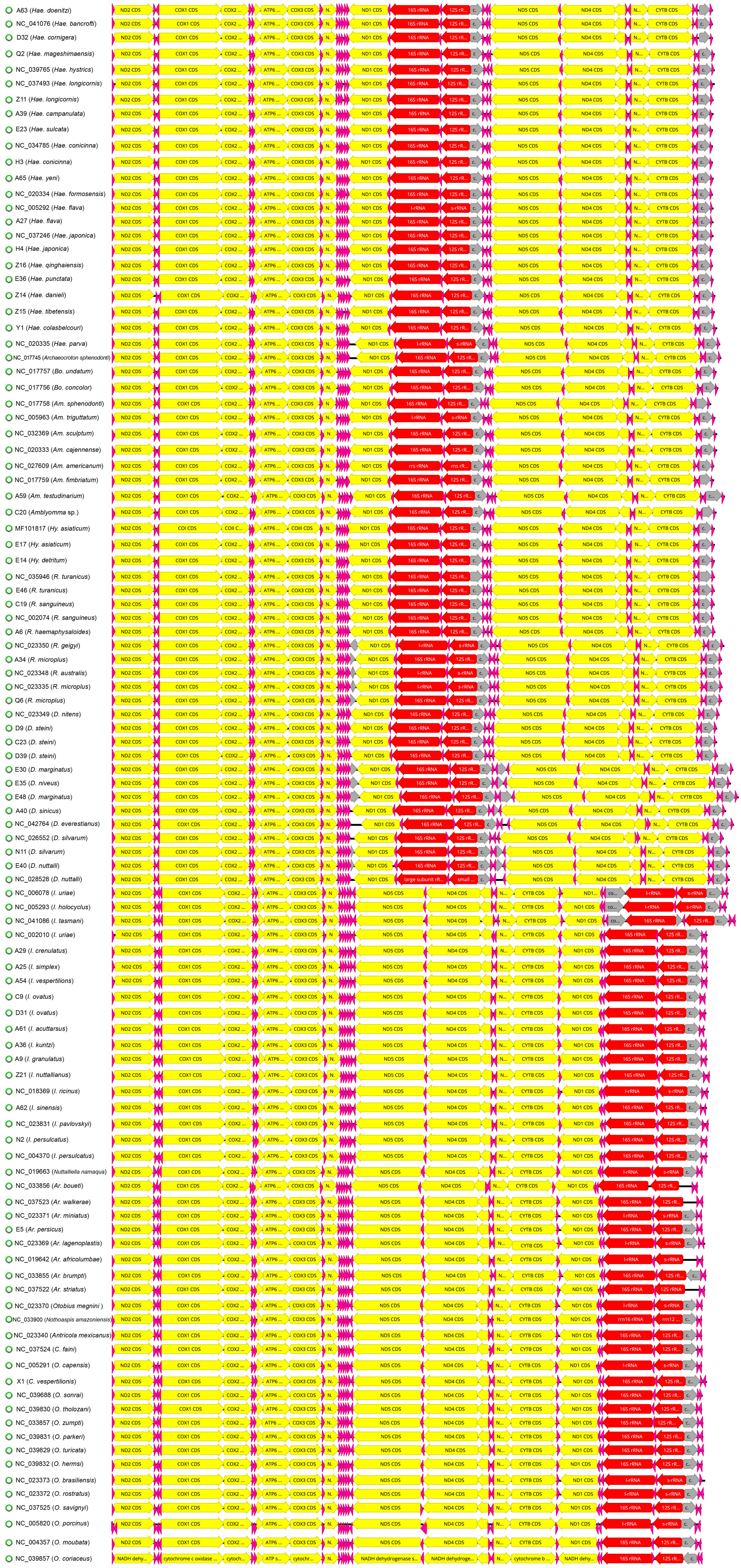
A63 (Hae. doenitzi)
NC_041076 (Hae. bancrofti)
D32 (Hae. cornigera)
Q2 (Hae. mageshimaensis)
NC_039765 (Hae. hystrics)
NC_037493 (Hae. longicornis)
Z11 (Hae. longicornis)
A39 (Hae. campanulata)
E23 (Hae. sulcata)
NC_034785 (Hae. conicinna)
H3 (Hae. conicinna)
A65 (Hae. yeni)
NC_020334 (Hae. formosensis)
NC_005292 (Hae. flava)
A27 (Hae. flava)
NC_037246 (Hae. japonica)
H4 (Hae. japonica)
Z16 (Hae. qinghaiensis)
E36 (Hae. punctata)
Z14 (Hae. danieli)
Z15 (Hae. tibetensis)
Y1 (Hae. colasbelcouri)
NC_020335 (Hae. parva)
NC_017745 (Archaeocroton sphenodonti)
NC_017757 (Bo. undatum)
NC_017756 (Bo. concolor)
NC_017758 (Am. sphenodonti)
NC_005963 (Am. triguttatum)
NC_032369 (Am. sculptum)
NC_020333 (Am. cajennense)
NC_027609 (Am. americanum)
NC_017759 (Am. fimbriatum)
A59 (Am. testudinarium)
C20 (Amblyomma sp.)
MF101817 (Hy. asiaticum)
E17 (Hy. asiaticum)
E14 (Hy. detritum)
NC_035946 (R. turanicus)
E46 (R. turanicus)
C19 (R. sanguineus)
NC_002074 (R. sanguineus)
A6 (R. haemaphysaloides)
NC_023350 (R. geigyi)
A34 (R. microplus)
NC_023348 (R. australis)
NC_023335 (R. microplus)
Q6 (R. microplus)
NC_023349 (D. nitens)
D9 (D. steini)
C23 (D. steini)
D39 (D. steini)
E30 (D. marginatus)
E35 (D. niveus)
E48 (D. marginatus)
A40 (D. sinicus)
NC_042764 (D. everestianus)
NC_026552 (D. silvarum)
N11 (D. silvarum)
E40 (D. nuttalli)
NC_028528 (D. nuttalli)
NC_006078 (I. uriae)
NC_005293 (I. holocyclus)
NC_041086 (I. tasmani)
NC_002010 (I. uriae)
A29 (I. crenulatus)
A25 (I. simplex)
A54 (I. vespertilions)
C9 (I. ovatus)
D31 (I. ovatus)
A61 (I. acuttarsus)
A36 (I. kuntzi)
A9 (I. granulatus)
Z21 (I. nuttallianus)
NC_018369 (I. ricinus)
A62 (I. sinensis)
NC_023831 (I. pavlovskyi)
N2 (I. persulcatus)
NC_004370 (I. persulcatus)
NC_019663 (Nuttalliella namaqua)
NC_033856 (Ar. boueti)
NC_037523 (Ar. walkerae)
NC_023371 (Ar. miniatus)
E5 (Ar. persicus)
NC_023369 (Ar. lagenoplastis)
NC_019642 (Ar. africolumbae)
NC_033855 (Ar. brumpti)
NC_037522 (Ar. striatus)
NC_023370 (Otobius megnini)
NC_033900 (Nothoaspis amazoniensis)
NC_023340 (Antricola mexicanus)
NC_037524 (C. faini)
NC_005291 (O. capensis)
X1 (C. vespertilionis)
NC_039688 (O. sonrai)
NC_039830 (O. tholozani)
NC_033857 (O. zumpti)
NC_039831 (O. parkeri)
NC_039829 (O. turicata)
NC_039832 (O. hermsi)
NC_023373 (O. brasiliensis)
NC_023372 (O. rostratus)
NC_037525 (O. savignyi)
NC_005820 (O. porcinus)
NC_004357 (O. moubata)
NC_039857 (O. coriaceus)

Additional file 1: Fig. S1. Gene arrangements in tick mitochondrial genomes across eight genera. There are 23 linear maps in nineteen species of *Haemaphysalis*, nine in *Amblyomma* species, two in *Bothriocroton* species, three in two *Hyalomma* species, 13 in ten *Rhipicephalus* species, 18 in 16 *Ixodes* species, one of Nuttalliellidae species and 26 linear maps of Argasid ticks. Each mitochondrial genome has 13 protein-coding genes, two ribosomal genes, 22 tRNA genes and misc-features varying with tick species. Mitochondrial gene arrangement and direction in *Haemaphysalis*, *Dermacentor*, *Amblyomma*, *Hyalomma*, *Rhipicephalus* and *Bothriocroton* are almost identical and those in Ixodes, Nuttalliellidae and Argasidae are almost identical. Protein-coding genes are denoted by yellow arrows, rRNA are denoted by red arrows, tRNA are denoted by pink arrows, and control regions are denoted by gray arrows. The direction of arrows indicated the direction of protein translation. Abbreviations are as follows: ND1=NADH dehydrogenase subunit 1, ND2=NADH dehydrogenase subunit 2, ND3=NADH dehydrogenase subunit 3, ND4= NADH dehydrogenase subunit 4, ND4L= NADH dehydrogenase subunit 4L, ND5=NADH dehydrogenase subunit 5, ND6= NADH dehydrogenase subunit 6, COX1= Cytochrome oxidase I, COX2= Cytochrome oxidase II, COX3= Cytochrome oxidase III, ATP8= ATPase8, ATP6= ATPase8, 16s rRNA= large ribosomal subunit, 12s rRNA= small ribosomal subunit, CYTB= Cytochrome b.

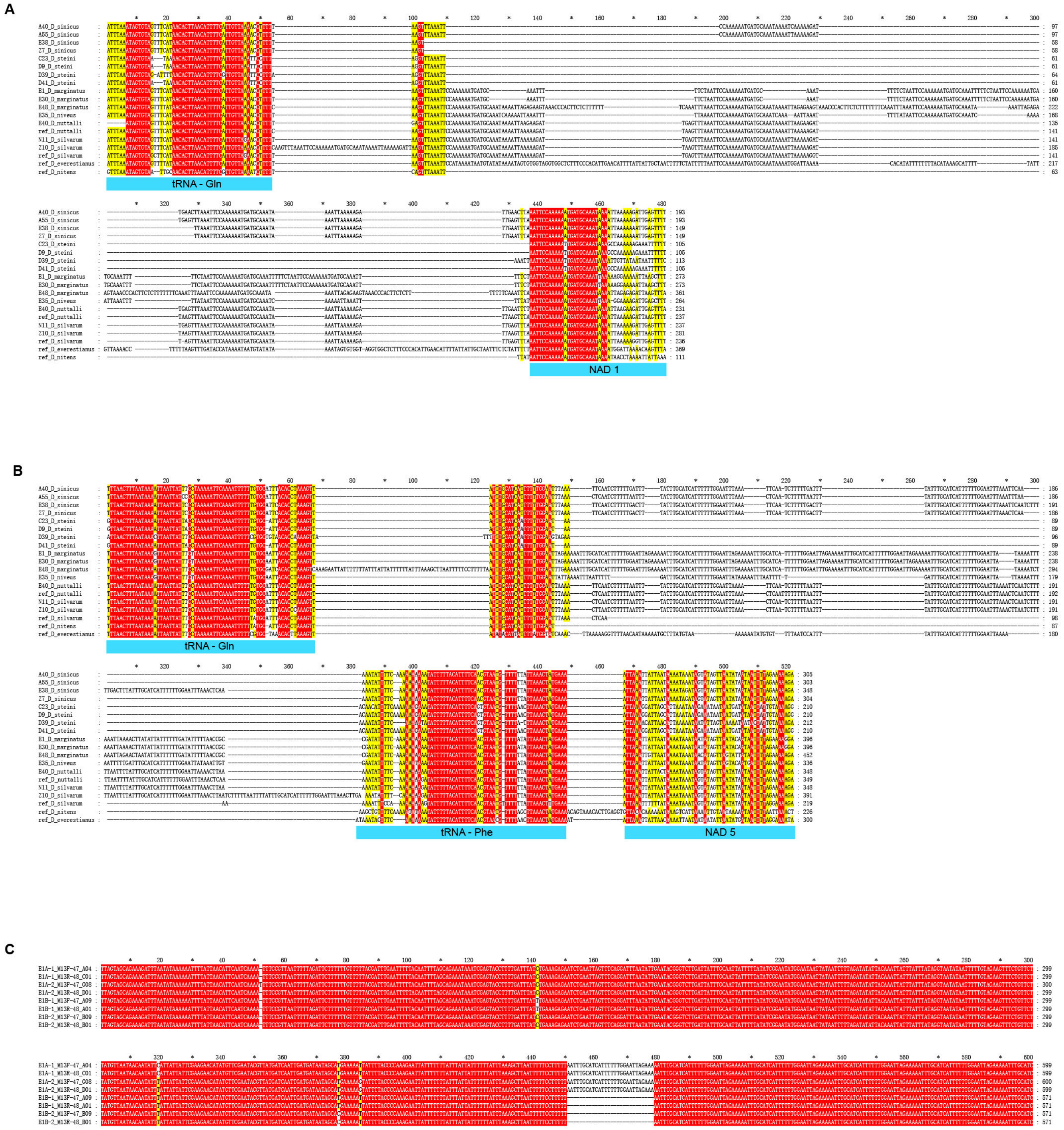

Additional file 1: Fig. S2. Sequence alignment of special tandem repeat regions in misc-features of *Dermacentor* mt genome. The first hypervariable region located between tRNA-Gln and NAD1 (A), and the second hypervariable region located tRNA-Gln between tRNA-Phe (B), and its cloning of sequencing of PCR products spanning the control region implied various copy numbers of short repeat sequences within the same *D. marginatus* E1 sample (C).

Additional file 1: Table S2. Special tandem repeat regions in misc-features of *Dermacentor* mt genome.

Strain	Species	Length (bp)	Misc feature			Tandem repeat region				Tandem repeat sequence
			From	To	Length	From	To	Length	Number	
N11	*D. silvarum*	15086	5837	5917	81	5827	5870	44	4	AGTTTAAATTCCAAAAAATGAT GCAAATAAAATTAAAAAGATTG
			5837	5917	81	5871	5914	44		
			5837	5917	81	5915	5958	44		
			5837	5917	81	5959	6002	44		
			9387	9551	165	9414	9457	44	3	CAATCTTTTTAATTTTATTTGCA TCATTTTTTGGAATTTAAACT
			9387	9551	165	9458	9501	44		
			9387	9551	165	9502	9545	44		
Z10	*D. silvarum*	15171	5838	5962	125	5827	5870	44	5	AAGTTTAAATTCCAAAAAATGA TGCAAATAAAATTAAAAAGATT
			5838	5962	125	5871	5914	44		
			5838	5962	125	5915	5958	44		
			5838	5962	125	5959	6002	44		
			5838	5962	125	6003	6046	44		
			9428	9636	209	9456	9499	44	4	AATCTTTTTAATTTTATTTGCAT CATTTTTTGGAATTTAAACTT
			9428	9636	209	9500	9543	44		
			9428	9636	209	9544	9587	44		
			9428	9636	209	9588	9631	44		
E1	*D. marginatus*	15178	5834	5995	162	5853	5880	28	6	TTTCTAATTCCAAAAAATGATGC AAATT
			5834	5995	162	5881	5908	28		
			5834	5995	162	5909	5936	28		
			5834	5995	162	5937	5964	28		
			5834	5995	162	5965	5992	28		
			5834	5995	162	5993	6020	28		
			9424	9635	212	9423	9450	28	5	ATTTGCATCATTTTTTGGAATTA GAAAA
			9424	9635	212	9451	9478	28		
			9424	9635	212	9479	9506	28		
			9424	9635	212	9507	9534	28		
			9424	9635	212	9535	9562	28		
A40	*D. sinicus*	14996	5831	5867	37	5824	5867	44	3.0	TTAAATTCCAAAAAATGATGCA AATAAAATCAAAAAGATTGAAC
			5831	5867	37	5868	5911	44		TTAAATTCCAAAAAATGATGCA AATAAAATTAAAAAGATTGAAC
			5831	5867	37	5912	5953	42		TTAAATTCCAAAAAATGATGCA AATAAAATTAAAAAGATTGA
			9334	9454	121	9331	9374	44	2.8	TTATTTGCATCATTTTTTGGAAT TTAAATTCAATCTTTTTGATT
			9334	9454	121	9375	9418	44		
			9334	9454	121	9419	9451	33		TTATTTGCATCATTTTTTGGAAT TTAAATTCAA
A55	*D. sinicus*	14997	5834	5870	37	5824	5867	44	3.0	AGTTTAAATTCCAAAAAATGAT GCAAATAAAATTAAAAAGATTG
			5834	5870	37	5868	5911	44		
			5834	5870	37	5912	5955	44		
			9338	9458	121	9335	9378	44	2	TTATTTGCATCATTTTTTGGAAT TTAAACTCAATCTTTTTAATT
			9338	9458	121	9379	9422	44		
Z7	*D. sinicus*	14947			-7	5824	5867	44	2	TTTAAATTCCAAAAAATGATGC AAATAAATTTAAAAAGATTGAA
					-7	5868	5911	44		TTTAAATTCCAAAAAATGATGC AAATAAAATTAAAAAAATTGAA
			9285	9408	124	9285	9328	44	2.8	TTATTTGCATCATTTTTTGGAAT TTAAATTCAATCTTTTTGACT
			9288	9408	121	9329	9372	44		
			9288	9408	121	9373	9405	33		TTATTTGCATCATTTTTTGGAAT TTAAACTCAA

E38	*D. sinicus*	14991			-7	5824	5867	44	2	TTTAAATTCCAAAAAAATGATGC AAATAAATTTAAAAAAGATTGAA
					-7	5868	5910	43		TTTAAATTCCAAAAAAATGATGC AAATAAAATTAAAAAAAATTGA
			9288	9452	165	9285	9328	44	3.8	TTATTTGCATCATTTTTTGGAAT TTAAATTCAATCTTTTTGACT
			9288	9452	165	9329	9372	44		
			9288	9452	165	9373	9416	44		
			9288	9452	165	9417	9449	33		TTATTTGCATCATTTTTTGGAAT TTAAACTCAA

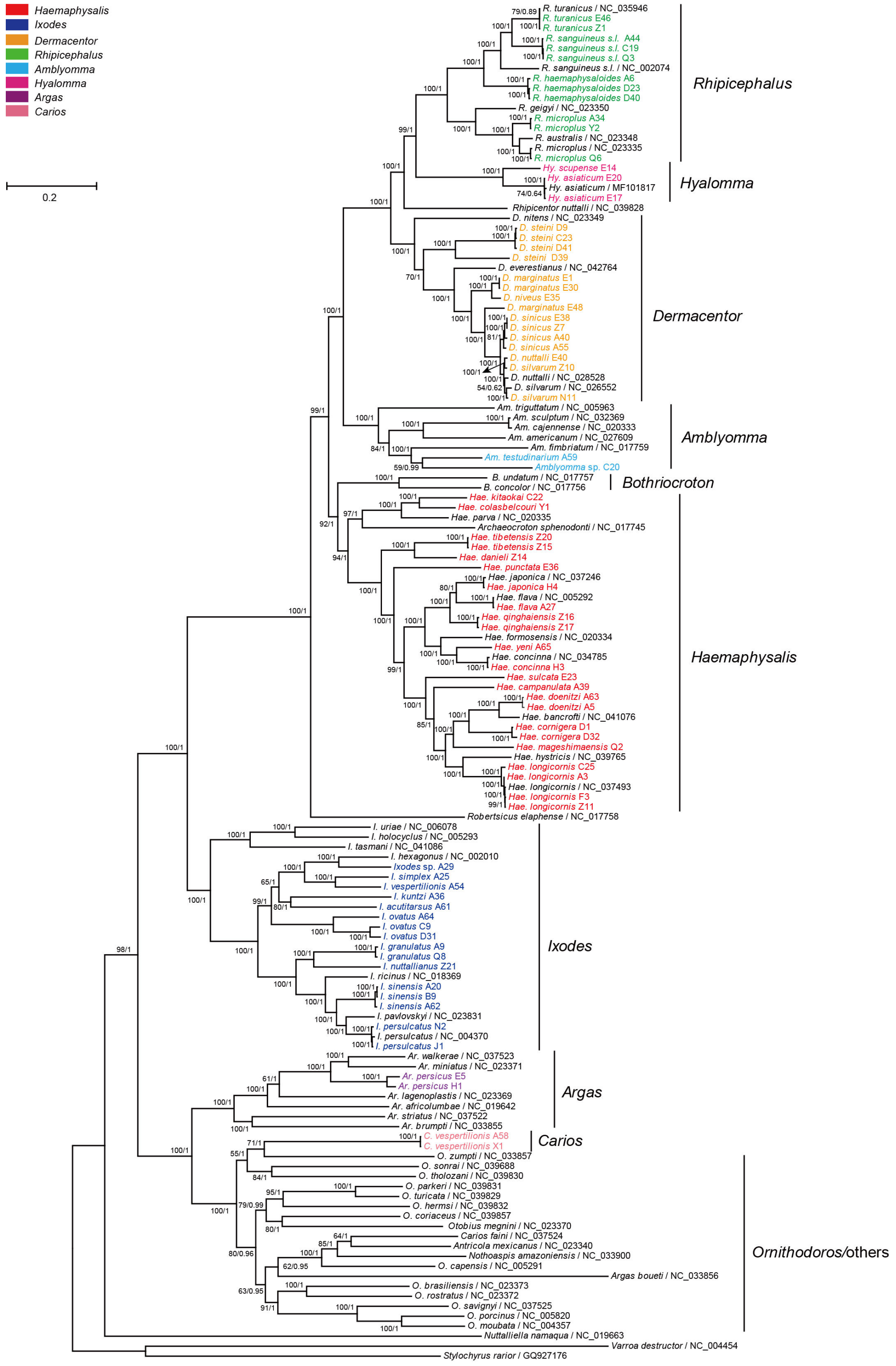

Additional file 1: Fig. S3. Detailed phylogenetic tree of ticks based on all 13 protein-coding genes and two rRNA gene inferred using both ML and Bayesian methods with two mites as the outgroup. Highly similar tree topologies were obtained. The trees are midpoint-rooted, and the scale bar represents the number of nucleotide substitutions per site. For clarity, the bootstrap values are shown on the left with posterior probability on the right for each node. Mt genomes identified in this study are marked colored font according to different tick genera, the name of each tick genus is shown aside.

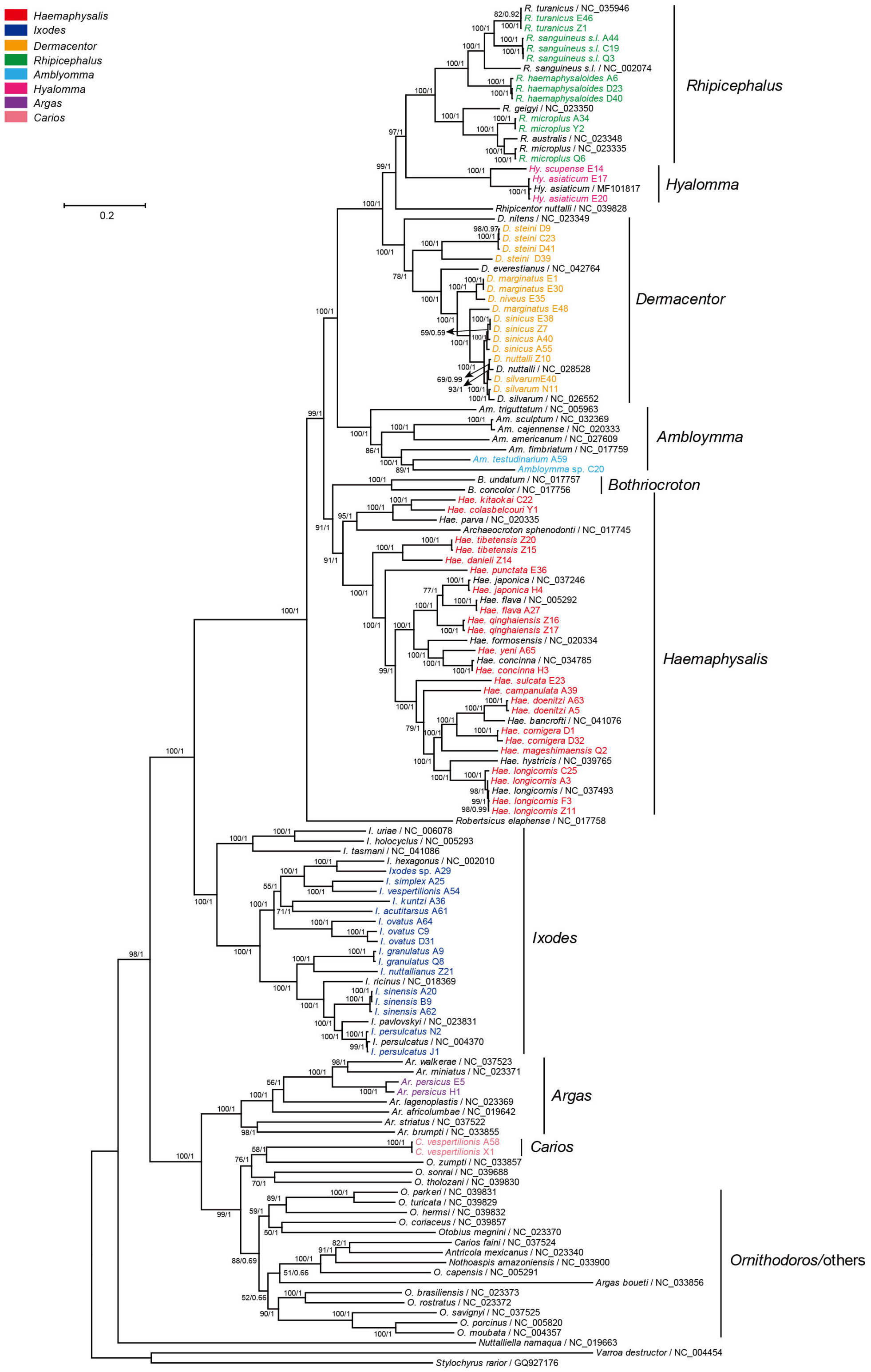

Additional file 1: Fig. S4. Detailed phylogenetic tree of ticks based on only 13 protein-coding genes estimated using both ML and Bayesian methods with two mites as the outgroup. Highly similar tree topologies were obtained. The trees are midpoint-rooted, and the scale bar represents the number of nucleotide substitutions per site. For each node, the bootstrap values are shown on the left with posterior probability on the right. Mt genomes identified in this study are marked colored font according to different tick genera, the name of each tick genus is shown aside.

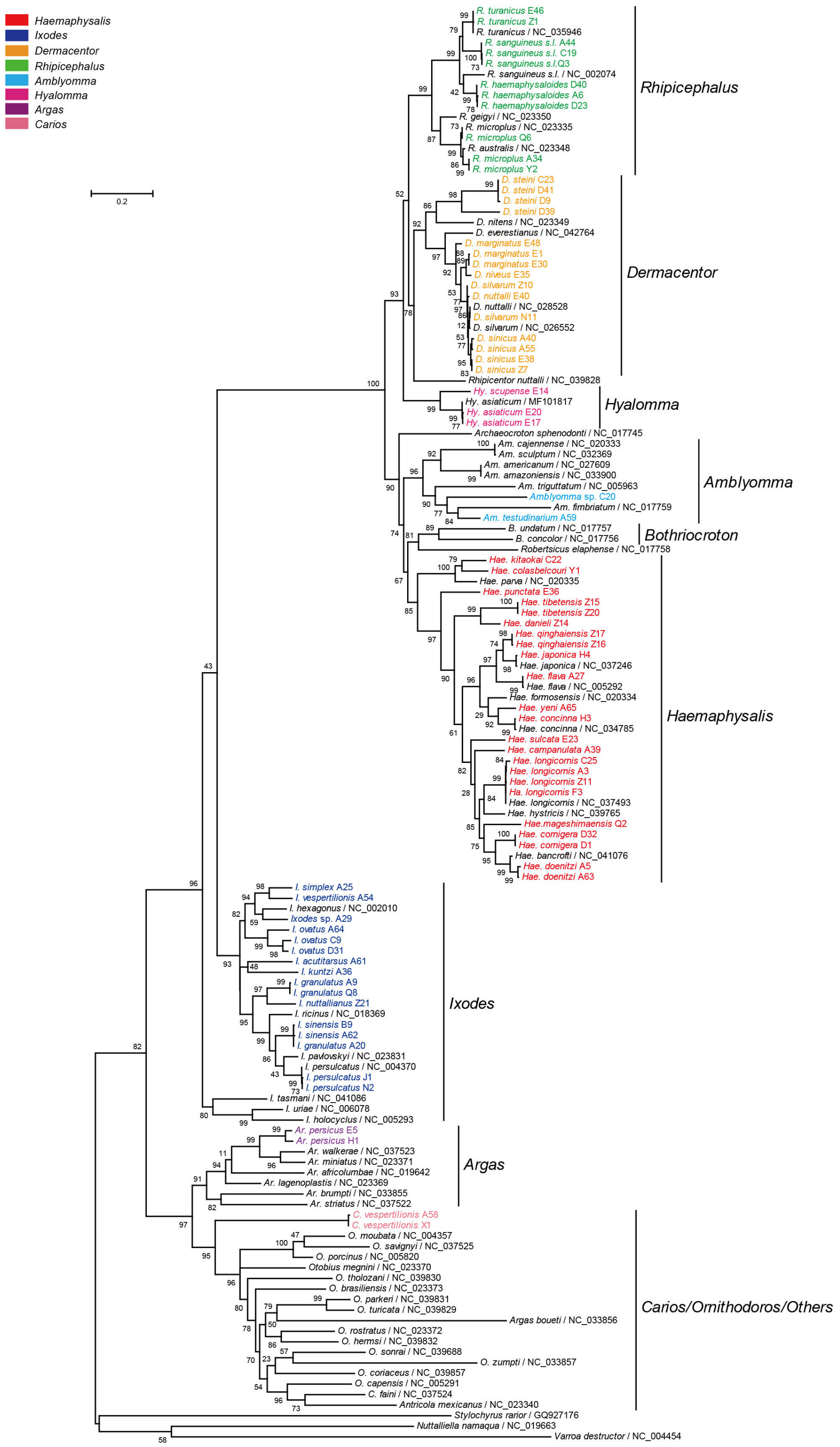

Additional file 1: Fig. S5. Detailed phylogenetic tree of ticks based on mt 12S rRNA gene estimated using ML methods. The tree is midpoint-rooted, and the scale bar represents the number of nucleotide substitutions per site. Mt 12S rRNA gene identified in this study are marked colored font according to different tick genera, the name of each tick genus is shown aside.

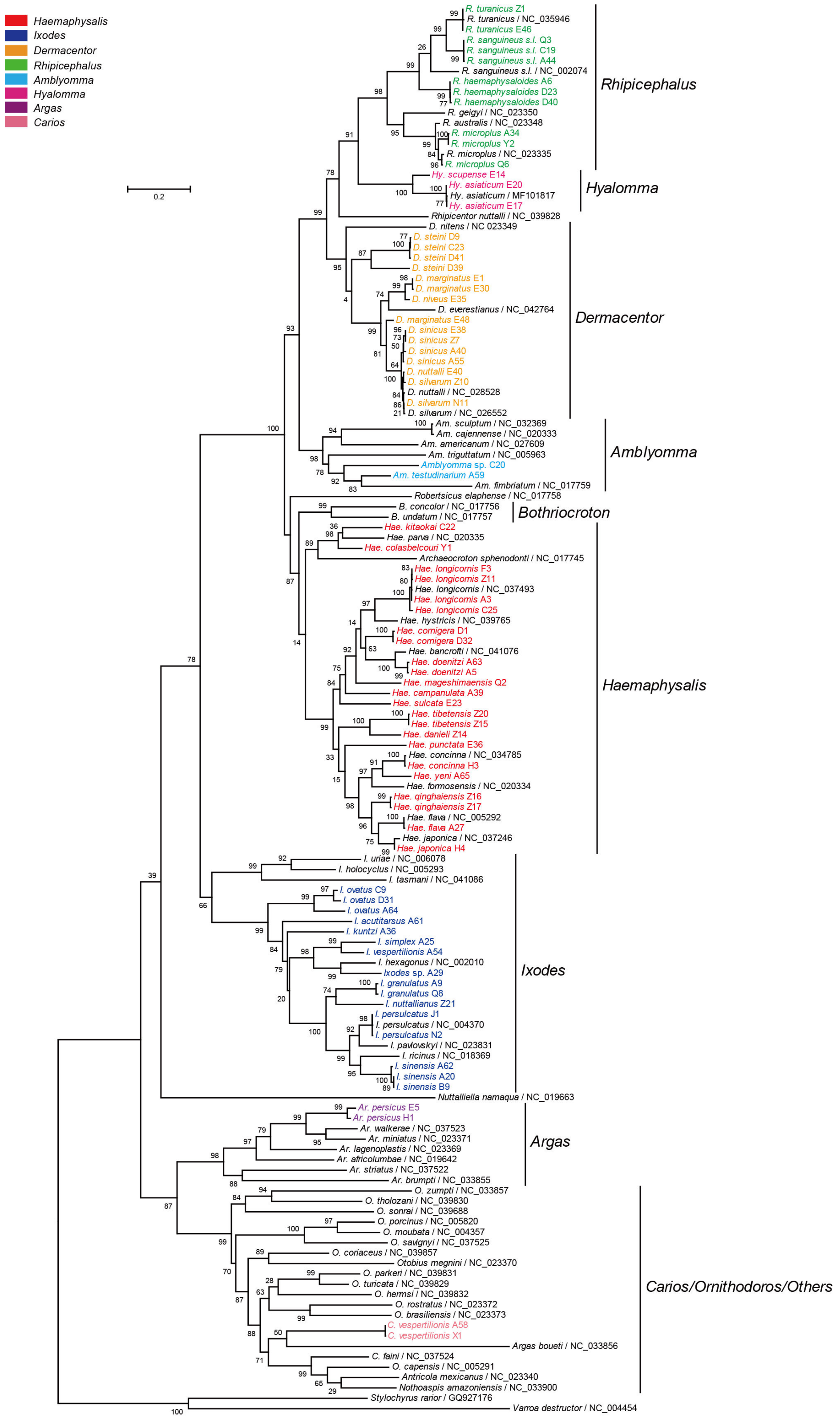

Additional file 1: Fig. S6. Detailed phylogenetic tree of ticks based on mt 16S rRNA gene estimated using ML methods. The tree is midpoint-rooted, and the scale bar represents the number of nucleotide substitutions per site. Mt 16S rRNA gene identified in this study are marked colored font according to different tick genera, the name of each tick genus is shown aside.

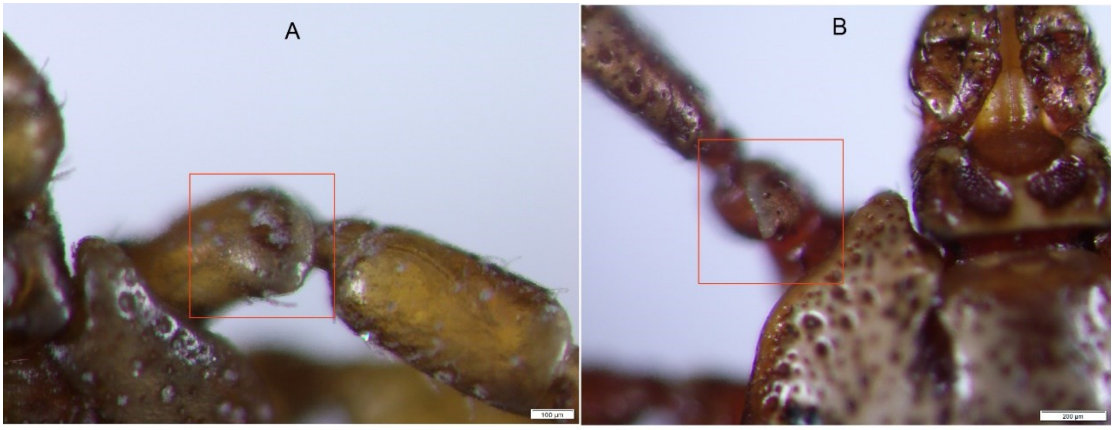

Additional file 1: Fig. S7. Differential diagnosis for *D. nuttalli* and *D. silvarum*. Adult trochanter I dorsal spur of *D. nuttalli* (A) is short, broad and blunt apically, while that of *D. silvarum* (B) is slightly long, pointed at the apex.

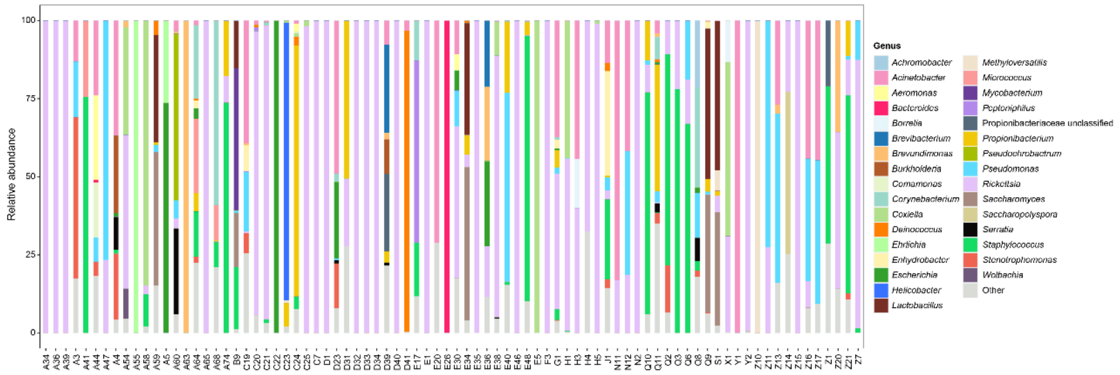

Additional file 1: Fig. S8. Relative abundance of bacterial and fungi at the level of genus based on metaphlan2 results.

Additional file 1: Table S3. Prevalence of tick-borne bacteria in ticks at the level of genus. A total of 43 tick associated bacterial species were identified from 54 libraries and 7 tick genera (with the exception of *Hyalomma*). Bracketed numbers denote numbers of bacteria identified in each bacterial group at the level of genera.

Pathogen(genus)	Tick host (This study)	No. of libraries	Libraries	No. of pathogens(seqs)
Rickettsia	*Rhipicephalus, Dermacentor, Amblyomma, Haemaphysalis, Ixodes, Argas, Carios*	26	A34[a], A36[e], A39[d], A54[e], A60[c], A65[d], C7[e], C20[c], C21[c], D1[d], D32[d], D40[a], E1[b], E30[b], E35[b], E38[b], E46[a], H1[g], H5[d], S1[d], G1[d], N2[e], X1[f], Y2[a], Z7[b], Z15[d]	14(27)
Rickettsiales unclassified	*Dermacentor*	3	A40[b], A55[b], E35[b]	1(3)
Coxiella	*Rhipicephalus, Dermacentor, Haemaphysalis, Ixodes, Carios, Argas*	40	A3[d], A4[d], A6[a], A27[d], A34[a], A44[a], A54[e], A55[b], A58[f], A64[e], A65[d], C19[a], C22[d], D1[d], D23[d], D31[e], D32[d], D39[b], D40[a], E1[b], E5[g], E23[d], E38[b], E46[a], H1[g], H3[d], H4[d], E48[b], N12[d], Q2[d], Q3[a], Q6[a], Q11[d], X1[g], Y2[a], Z1[a], Z11[d], Z14[d], Z15[d], Z16[d]	26(41)
Borrelia	*Carios*	1	X1[f]	1(1)
Wolbachia	*Ixodes*	1	A54[e]	1(1)
Tatol	7	54	—	43(73)

a-g: *Rhipicephalus, Dermacentor, Amblyomma, Haemaphysalis, Ixodes, Carios, Argas*.

Additional file 1: Table S4. The genetic similarity of bacterial strains identified in this study with its closest reference sequence.

Taxonomy	Pathogen/endosymbiont in this study	The closest reference	Genetic similarity
Rickettsia	*Rickettsia africae* A54a	*Rickettsia africae* ESF-5 (NC_012633)	99.865%
Rickettsia	*Rickettsia africae* E46	*Rickettsia africae* ESF-5 (NC_012633)	99.318%
Rickettsia	*Rickettsia* endosymbiont *of Argas persicus* H1	*Rickettsia bellii* OSU 85-389 (NC_009883)	92.164%
Rickettsia	*Rickettsia* endosymbiont *of Carios vespertilionis* X1	*Rickettsia* endosymbiont *of Culicoides newsteadi* strain RiCNE (NZ_MWZE01000001)	85.969%
Rickettsia	*Rickettsia* endosymbiont *of Haemaphysalis megaspinosa* S1	*Rickettsia fournieri* strain AUS118 Argas lagenoplastis (NZ_LT978484)	99.455%
Rickettsia	*Rickettsia* endosymbiont *of Ixodes persulcatus* H5	*Rickettsia canadensis* strain CA410 (NC_016929)	97.918%
Rickettsia	*Rickettsia* endosymbiont *of Ixodes persulcatus* N2	*Rickettsia canadensis* strain McKiel (NC_009879)	97.776%
Rickettsia	*Rickettsia* endosymbiont *of Ixodes vespertilionis* A54b	*Rickettsia africae* ESF-5 (NC_012633)	90.828%
Rickettsia	*Rickettsia fournieri* A34	*Rickettsia fournieri* strain AUS118 Argas lagenoplastis (NZ_LT978484)	99.728%
Rickettsia	*Rickettsia fournieri* A65	*Rickettsia fournieri* strain AUS118 Argas lagenoplastis (NZ_LT978484)	99.591%
Rickettsia	*Rickettsia fournieri* C20	*Rickettsia fournieri* strain AUS118 Argas lagenoplastis (NZ_LT978484)	99.728%
Rickettsia	*Rickettsia fournieri* C21	*Rickettsia fournieri* strain AUS118 Argas lagenoplastis (NZ_LT978484)	99.728%
Rickettsia	*Rickettsia fournieri* G1	*Rickettsia fournieri* strain AUS118 Argas lagenoplastis (NZ_LT978484)	99.728%
Rickettsia	*Rickettsia fournieri* Y2	*Rickettsia fournieri* strain AUS118 Argas lagenoplastis (NZ_LT978484)	99.728%
Rickettsia	*Rickettsia fournieri* Z15	*Rickettsia fournieri* strain AUS118 Argas lagenoplastis (NZ_LT978484)	99.864%
Rickettsia	*Rickettsia heilongjiangensis* A39	*Rickettsia heilongjiangensis* 054 (NC_015866)	100.000%
Rickettsia	*Rickettsia heilongjiangensis* D1	*Rickettsia heilongjiangensis* 054 (NC_015866)	100.000%
Rickettsia	*Rickettsia heilongjiangensis* D32	*Rickettsia heilongjiangensis* 054 (NC_015866)	100.000%
Rickettsia	*Rickettsia helvetica* A36	*Rickettsia helvetica* C9P9 (NZ_CM001467)	100.000%
Rickettsia	*Rickettsia monacensis* C7	*Rickettsia monacensis* IrRMunich (NZ_LN794217)	100.000%
Rickettsia	*Rickettsia raoultii E1*	*Rickettsia raoultii* strain Khabarovsk (NZ_CP010969)	100.000%
Rickettsia	*Rickettsia raoultii* E30	*Rickettsia raoultii* strain Khabarovsk (NZ_CP010969)	100.000%
Rickettsia	*Rickettsia raoultii* E35	*Rickettsia raoultii* strain Khabarovsk (NZ_CP010969)	100.000%
Rickettsia	*Rickettsia rhipicephali* D40	*Rickettsia rhipicephali* strain HJ5 (NZ_CP013133)	99.592%
Rickettsia	*Rickettsia sibirica* E38	*Rickettsia sibirica* 246 (NZ_AABW01000001)	100.000%
Rickettsia	*Rickettsia sibirica* Z7	*Rickettsia sibirica* 246 (NZ_AABW01000001*)*	100.000%
Rickettsia	*Rickettsia tamurae* A60	*Rickettsia tamurae* (NZ_CCMG01000009)	100.000%
Rickettsiales unclassfied	*Rickettsiales* endosymbiont *of Dermacentor* Hubei A40	*Rickettsiales bacterium* Ac37b (NZ_CP009217)	86.505%
Rickettsiales unclassfied	*Rickettsiales* endosymbiont *of Dermacentor* Hubei A55	*Rickettsiales bacterium* Ac37b (NZ_CP009217)	86.677%
Rickettsiales unclassfied	*Rickettsiales* endosymbiont *of Dermacentor* Xinjiang E35	*Rickettsiales bacterium* Ac37b (NZ_CP009217)	86.677%
Wolbachia	*Wolbachia* endosymbiont *of Ixodes vespertilionis* A54	*Wolbachia pipientis* strain FL2016 (NZ_NWVK01000027)	95.369%
Wolbachia	*Wolbachia* endosymbiont *of Ixodes vespertilionis* A54	*Wolbachia* endosymbiont *of Drosophila simulans* wHa (NC_021089)	95.276%
Coxiella	*Coxiella burnetii* E38b	*Coxiella burnetii* MSU Goat Q177 (NZ_CP018150)	99.104%
Coxiella	*Coxiella* endosymbiont *of Argas persicus* E5	*Coxiella* endosymbiont *of Ornithodoros amblus* (KP985448)	99.104%
Coxiella	*Coxiella* endosymbiont *of Argas persicus* H1	*Coxiella* endosymbiont *of Ornithodoros amblus* (KP985448)	94.453%
Coxiella	*Coxiella* endosymbiont *of Carios vespertilionis* A54	*Coxiella* endosymbiont *of Ornithodoros amblus* (KP985448)	96.315%
Coxiella	*Coxiella* endosymbiont *of Carios vespertilionis* A58	*Coxiella* endosymbiont *of Ornithodoros amblus* (KP985448)	96.315%
Coxiella	*Coxiella* endosymbiont *of Carios vespertilionis* X1	*Coxiella* endosymbiont *of Ornithodoros amblus* (KP985448)	96.315%
Coxiella	*Coxiella* endosymbiont *of Dermacentor marginatus* E1	*Coxiella* endosymbiont *of Dermacentor silvarum* (KP985490)	99.102%
Coxiella	*Coxiella* endosymbiont *of Dermacentor marginatus* E48	*Coxiella* endosymbiont *of Dermacentor silvarum* (KP985490)	100.000%
Coxiella	*Coxiella* endosymbiont *of Dermacentor sinicus* A55	*Coxiella* endosymbiont *of Dermacentor silvarum* (KP985490)	100.000%
Coxiella	*Coxiella* endosymbiont *of Dermacentor sinicus* E38a	*Coxiella* endosymbiont *of Dermacentor silvarum* (KP985490)	100.000%
Coxiella	*Coxiella* endosymbiont *of Dermacentor steini* D39	*Coxiella* endosymbiont *of Dermacentor silvarum* (KP985490)	87.481%
Coxiella	*Coxiella* endosymbiont *of Haemaphysalis concinna* H3	*Coxiella* endosymbiont *of Rhipicephalus pusillus* (KP985519)	93.502%
Coxiella	*Coxiella* endosymbiont *of Haemaphysalis concinna* N12	*Coxiella* endosymbiont *of Rhipicephalus pusillus* (KP985519)	93.502%
Coxiella	*Coxiella* endosymbiont *of Haemaphysalis cornigera* D1	*Coxiella* endosymbiont *of Ixodes uriae* (KP985494)	88.534%
Coxiella	*Coxiella* endosymbiont *of Haemaphysalis cornigera* D32	*Coxiella* endosymbiont *of Ixodes uriae* (KP985494)	88.534%
Coxiella	*Coxiella* endosymbiont *of Haemaphysalis danieli* Z14	*Coxiella* endosymbiont *of Rhipicephalus bursa* (KP985509)	93.502%
Coxiella	*Coxiella* endosymbiont *of Haemaphysalis flava* A27	*Coxiella* endosymbiont *of Ornithodoros erraticus* (KP985455)	91.602%

Coxiella	*Coxiella* endosymbiont *of Haemaphysalis japonica* H4	*Coxiella* endosymbiont *of Ornithodoros erraticus* (KP985455)	91.652%
Coxiella	*Coxiella* endosymbiont *of Haemaphysalis kitaokai* C22	*Coxiella* endosymbiont *of Ornithodoros maritimus* (KP985461)	79.532%
Coxiella	*Coxiella* endosymbiont *of Haemaphysalis lagrangei* Q10	*Coxiella* endosymbiont *of Ixodes uriae* (KP985494)	89.575%
Coxiella	*Coxiella* endosymbiont *of Haemaphysalis longicornis* A3	*Coxiella* endosymbiont *of Ixodes uriae* (KP985494)	90.615%
Coxiella	*Coxiella* endosymbiont *of Haemaphysalis longicornis* A4	*Coxiella* endosymbiont *of Ixodes uriae* (KP985494)	90.615%
Coxiella	*Coxiella* endosymbiont *of Haemaphysalis longicornis* Z11	*Coxiella* endosymbiont *of Ixodes uriae* (KP985494)	90.615%
Coxiella	*Coxiella* endosymbiont *of Haemaphysalis mageshimaensis* Q2	*Coxiella* endosymbiont *of Ixodes uriae* (KP985494)	89.575%
Coxiella	*Coxiella* endosymbiont *of Haemaphysalis qinghaiensis* Z16	*Coxiella* endosymbiont *of Ornithodoros erraticus* (KP985455)	91.652%
Coxiella	*Coxiella* endosymbiont *of Haemaphysalis sulcata* E23	*Coxiella* endosymbiont *of Ixodes uriae* (KP985494)	90.671%
Coxiella	*Coxiella* endosymbiont *of Haemaphysalis tibetensis* Z15	*Coxiella* endosymbiont *of Rhipicephalus bursa* (KP985509)	96.358%
Coxiella	*Coxiella* endosymbiont *of Haemaphysalis yeni* A65	*Coxiella* endosymbiont *of Ornithodoros erraticus* (KP985455)	95.419%
Coxiella	*Coxiella* endosymbiont *of Ixodes ovatus* A64	*Coxiella* endosymbiont *of Ornithodoros amblus* (KP985448)	83.119%
Coxiella	*Coxiella* endosymbiont *of Ixodes ovatus* D31	*Coxiella* endosymbiont *of Ornithodoros amblus* (KP985448)	85.318%
Coxiella	*Coxiella* endosymbiont *of Rhipicephalus haemaphysaloides* A6	*Candidatus* Coxiella mudrowiae (CP011126)	99.099%
Coxiella	*Coxiella* endosymbiont *of Rhipicephalus haemaphysaloides* D23	*Candidatus* Coxiella mudrowiae (CP011126)	99.099%
Coxiella	*Coxiella* endosymbiont *of Rhipicephalus haemaphysaloides* D40	*Candidatus* Coxiella mudrowiae (CP011126)	99.099%
Coxiella	*Coxiella* endosymbiont *of Rhipicephalus microplus* A34	*Coxiella* endosymbiont *of Rhipicephalus microplus* (NZ_DLUJ01000017)	99.104%
Coxiella	*Coxiella* endosymbiont *of Rhipicephalus microplus* Q6	*Coxiella* endosymbiont *of Rhipicephalus australis* (KP985507)	100.000%
Coxiella	*Coxiella* endosymbiont *of Rhipicephalus microplus* Y2	*Coxiella* endosymbiont *of Rhipicephalus microplus* (NZ_DLUJ01000017)	99.104%
Coxiella	*Coxiella* endosymbiont *of Rhipicephalus sanguineus* A44	*Coxiella* endosymbiont *of Rhipicephalus sanguineus* (MK119208)	100.000%
Coxiella	*Coxiella* endosymbiont *of Rhipicephalus sanguineus* C19	*Coxiella* endosymbiont *of Rhipicephalus sanguineus* (MK119208)	100.000%
Coxiella	*Coxiella* endosymbiont *of Rhipicephalus sanguineus* Q3	*Coxiella* endosymbiont *of Rhipicephalus sanguineus* (MK119208)	100.000%
Coxiella	*Coxiella* endosymbiont *of Rhipicephalus turanicus* E46	*Candidatus* Coxiella mudrowiae (CP011126)	100.000%
Coxiella	*Coxiella* endosymbiont *of Rhipicephalus turanicus* Z1	*Candidatus* Coxiella mudrowiae (CP011126)	100.000%
Borrelia	*Borrelia henanensis* X1	*Borrelia duttonii* CR2A (NZ_AZIT01000001)	98.911%
Borrelia	*Borrelia henanensis* X1	*Borrelia crocidurae Achema* (NC_017808)	98.860%
Borrelia	*Borrelia henanensis* X1	*Borrelia recurrentis* A1 (NC_011244)	98.811%
Borrelia	*Borrelia henanensis* X1	*Borrelia duttonii* Ly (NC_011229)	98.811%
Borrelia	*Borrelia henanensis* X1	*Borrelia crocidurae* DOU (NZ_CP004267)	98.761%
Borrelia	*Borrelia henanensis* X1	*Borrelia hispanica* CRI (NZ_AYOU01000164)	97.960%

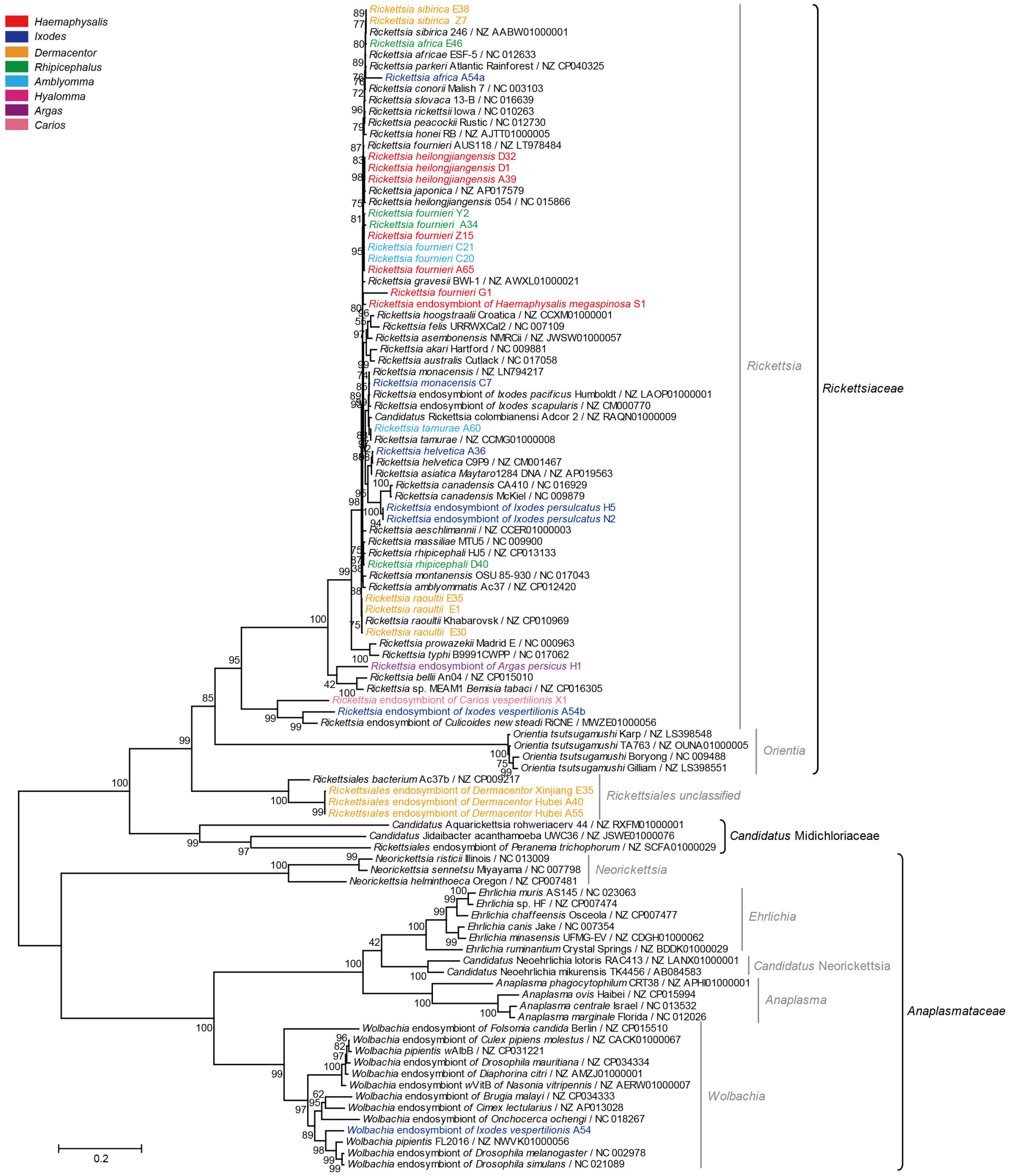

Additional file 1: Fig. S9. ML phylogenetic tree of the order Rickettsiales based on the groEL gene. The tree is midpoint-rooted, and the scale bar represents the number of nucleotide substitutions per site. The strains identified here are marked by colored font according to different tick genera, and reference sequences are represented by black font with corresponding accession number nearby. Each bacterial group at genus level of is denoted by gray font and a vertical line, while bacterial group at subfamily level is denoted by black font and a square bracket.

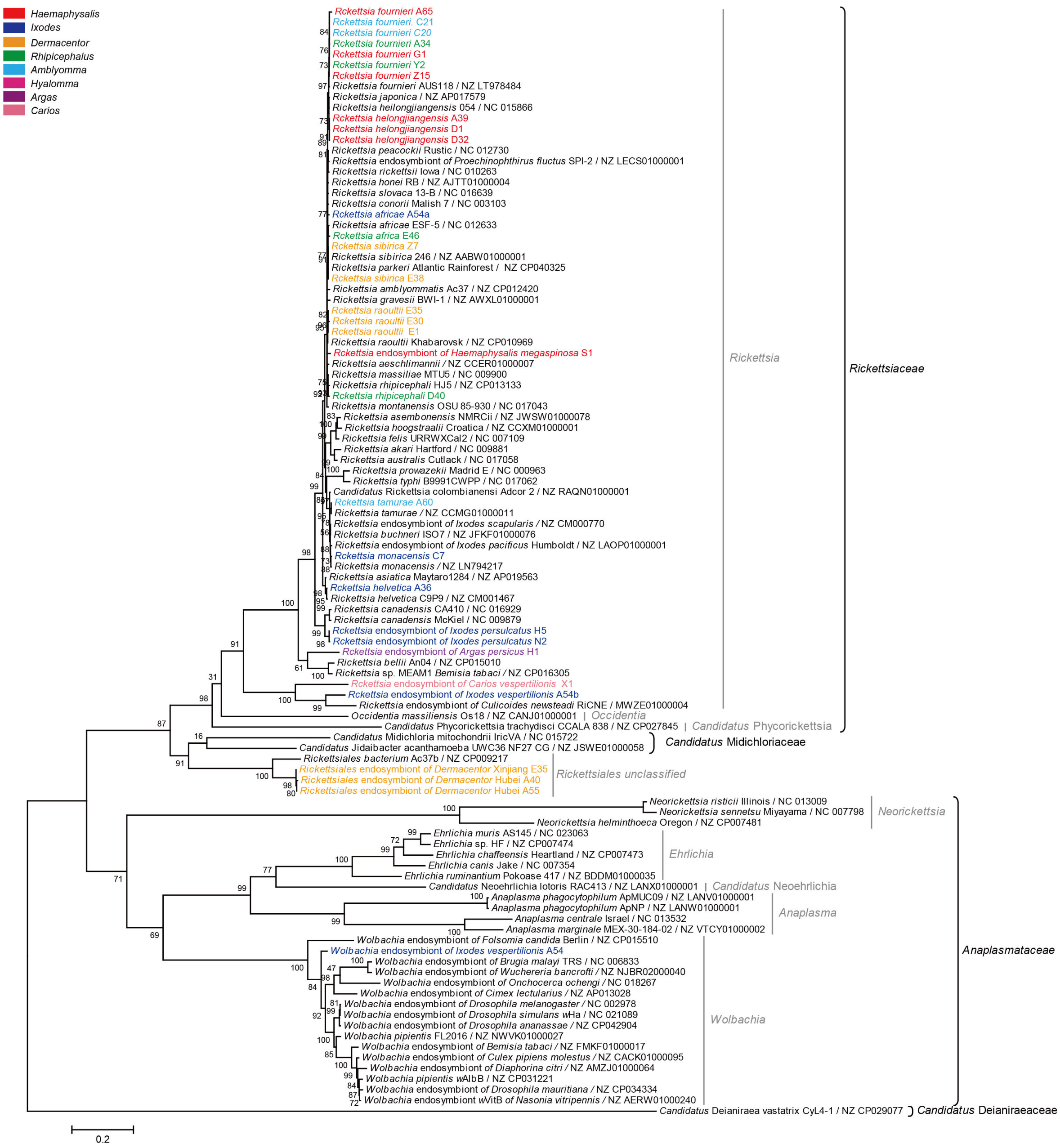

Additional file 1: Fig. S10. ML phylogenetic tree of the order Rickettsiales based on the gltA gene. The tree is midpoint-rooted, and the scale bar represents the number of nucleotide substitutions per site. The strains identified here are marked by colored font according to different tick genera, and reference sequences are represented by black font with corresponding accession number nearby. Each bacterial group at genus level is denoted by gray font and vertical line, while bacterial group at subfamily level is denoted by black font and square bracket.

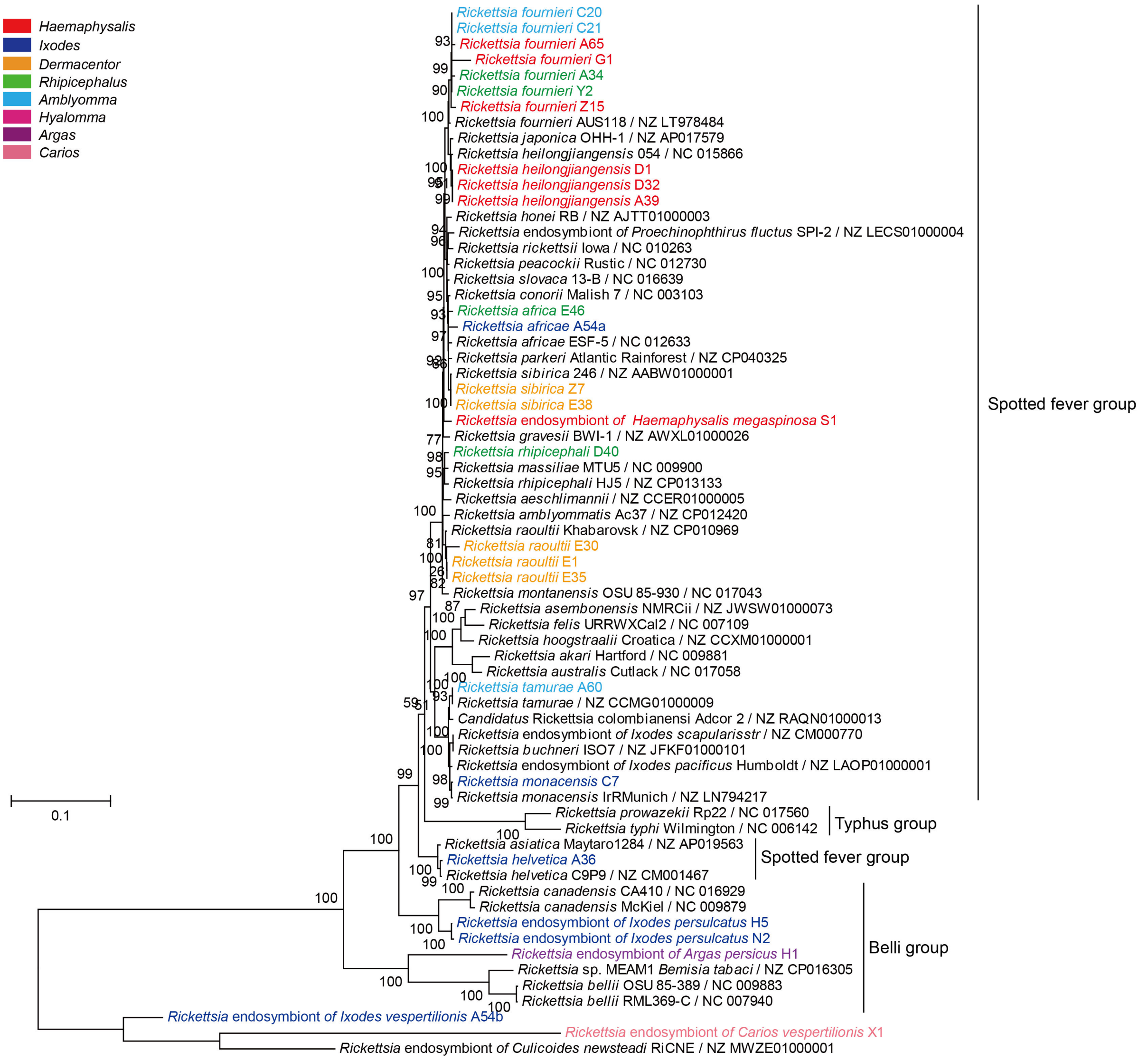

Additional file 1: Fig. S11. ML phylogenetic tree of the genus *Rickettsia* based on the six conserved housekeeping genes, including the atpD, coxB, ftsZ, gltA, groEL and sucA genes. Individual genes were first aligned and then concatenated to form super-alignment for phylogenetic analysis. The tree is midpoint-rooted, and the scale bar represents the number of nucleotide substitutions per site. The strains identified here are marked by colored font according to different tick genera, and reference sequences are represented by black font with corresponding accession number nearby. The Rickettsiae collected here could further assigned into three groups: Spotted fever group (SFG), Typhus group (TG), and Belli group (BG).

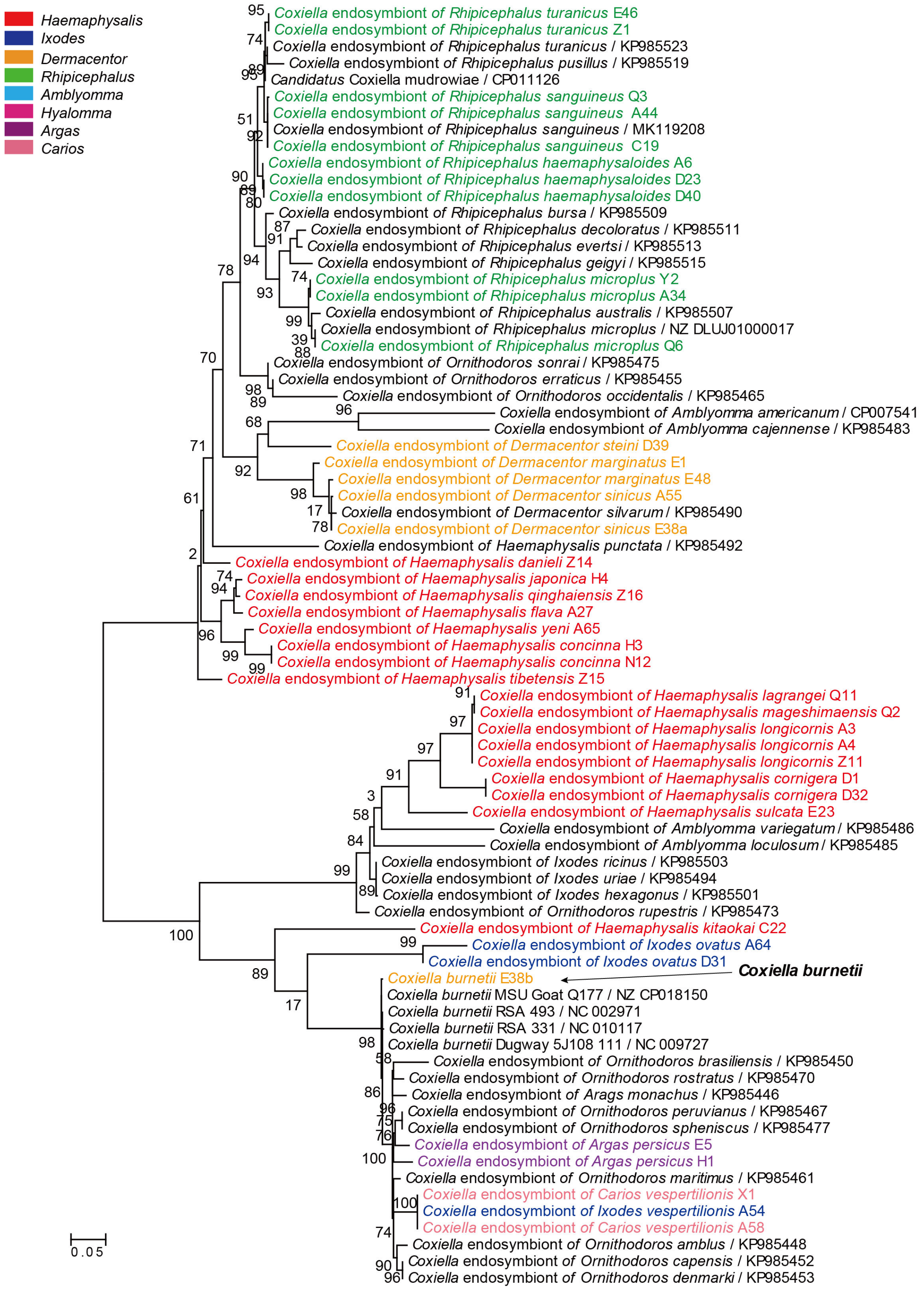

Additional file 1: Fig. S12. ML phylogenetic tree of the genus *Coxiella* based on the groEL gene. The tree is midpoint-rooted, and the scale bar represents the number of nucleotide substitutions per site. The strains identified here are marked by colored font according to different tick genera, and reference sequences are represented by black font with corresponding accession number nearby. A significant clustering of *Coxiella* genetic diversity at the host general level was observed, and the position of *C. burnetii* was highlighted by an arrow.

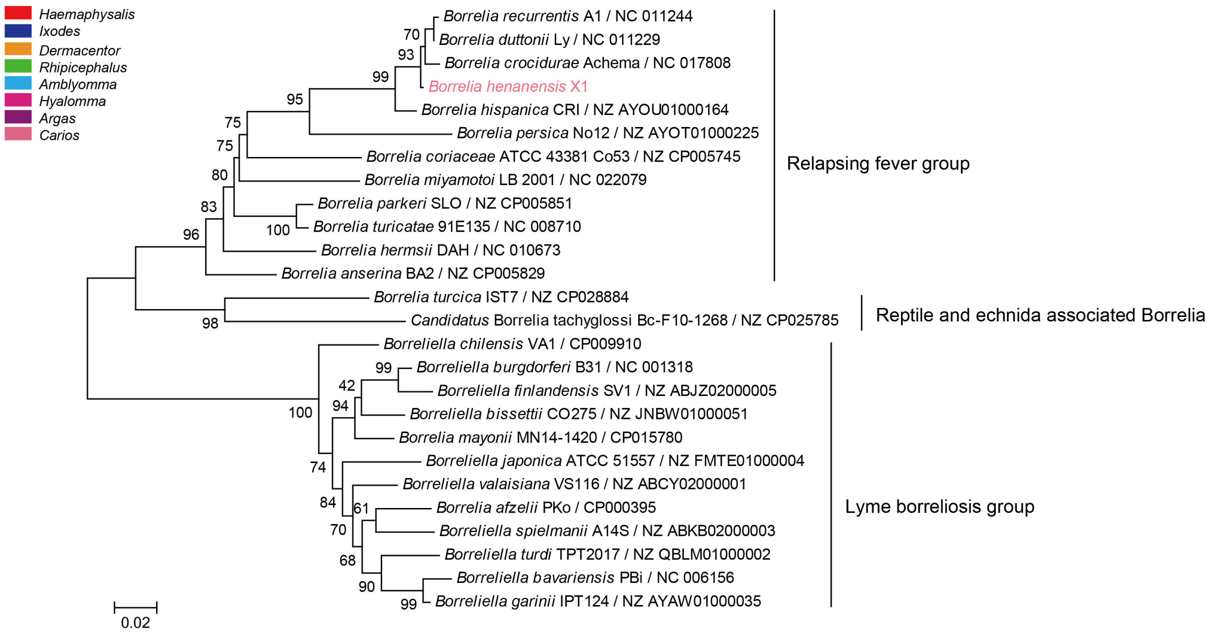

Additional file 1: Fig. S13. ML phylogenetic tree of the genus *Borrelia* based on the flaB. The tree is midpoint-rooted, and the scale bar represents the number of nucleotide substitutions per site. The trees are midpoint-rooted, and the scale bar represents the number of nucleotide substitutions per site. The *B. henanensis* strain X1 identified here is marked in pink font according to its tick genus (*Carios*), and reference sequences are represented by black font with corresponding accession number nearby. The *Borrelia* collected here could be assigned into three groups: The Relapsing fever group (RFG), Reptile and echidna associated *Borrelia* (REB), and Lyme borreliosis group (LB).

Additional file 1: Table S5. Multiple regression of bacteria genetic distance against tick genetic distance and geographic distance shows that bacterial genetic diversity was mainly structured by tick genetic distance rather than geographical distribution.

	Variable	Correlation coefficient	P value
Rickettsia	Tick	1.51E-01	0.0006
	Geography	-3.20E-06	0.3489
Coxiella	Tick	2.99E-02	0.0001
	Geography	2.72E-07	0.9416
